# AI Applied to Cardiac Magnetic Resonance for Precision Medicine in Coronary Artery Disease: A Systematic Review

**DOI:** 10.3390/jcdd12090345

**Published:** 2025-09-09

**Authors:** Cristina Jiménez-Jara, Rodrigo Salas, Rienzi Díaz-Navarro, Steren Chabert, Marcelo E. Andia, Julián Vega, Jesús Urbina, Sergio Uribe, Tetsuro Sekine, Francesca Raimondi, Julio Sotelo

**Affiliations:** 1 School of Biomedical Engineering, Universidad de Valparaíso, Valparaíso 2362905, Chile; maria.jimenezj@postgrado.uv.cl (C.J.-J.); rodrigo.salas@uv.cl (R.S.); steren.chabert@uv.cl (S.C.); 2 Cardiovascular Physiology Laboratory, Department of Medicine, School of Medicine, Universidad de Valparaíso, Viña del Mar 2340000, Chile; rienzi.diaz@uv.cl; 3 Millennium Institute for Intelligent Healthcare Engineering—iHealth, Valparaíso 2362905, Chile; meandia@uc.cl; 4 Center of Interdisciplinary Biomedical and Engineering Research for Health—MEDING, Universidad de Valparaíso, Valparaíso 2362905, Chile; 5 Biomedical Imaging Center and Department of Radiology, School of Medicine, Pontificia Universidad Católica de Chile, Santiago 8330023, Chile; 6 Department of Radiology, Complejo Asistencial Dr. Sótero del Río, Santiago 8207257, Chile; julianvega@gmail.com (J.V.); jjesus.urbina@gmail.com (J.U.); 7 Chilean Institute of Cardiac Imaging —INCIC, Santiago 8320000, Chile; 8 Department of Radiology, Clínica BUPA Santiago, Santiago 8240004, Chile; 9 Department of Medical Imaging and Radiation Sciences, Monash University, Melbourne 3800, Australia; sergio.uribe@monash.edu; 10 Department of Radiology, Nippon Medical School Musashi Kosugi Hospital, Kanagawa 211-8533, Japan; tetsuro.sekine@gmail.com; 11 Congenital Heart Disease Unit, Papa Giovanni XXXIII Hospital, 24127 Bergamo, Italy; 12 Departamento de Informática, Universidad Técnica Federico Santa María, Santiago 8940897, Chile

**Keywords:** AI, systematic review, artificial intelligence, CAD, coronary artery disease, CMR, cardiac magnetic resonance

## Abstract

Cardiac magnetic resonance (CMR) imaging has become a key tool in evaluating myocardial injury secondary to coronary artery disease (CAD), providing detailed assessments of cardiac morphology, function, and tissue composition. The integration of artificial intelligence (AI), including machine learning and deep learning techniques, has enhanced the diagnostic capabilities of CMR by automating segmentation, improving image interpretation, and accelerating clinical workflows. Radiomics, through the extraction of quantitative imaging features, complements AI by revealing sub-visual patterns relevant to disease characterization. This systematic review analyzed AI applications in CMR for CAD. A structured search was conducted in MEDLINE, Web of Science, and Scopus up to 17 March 2025, following PRISMA guidelines and quality-assessed with the CLAIM checklist. A total of 106 studies were included: 46 on classification, 19 using radiomics, and 41 on segmentation. AI models were used to classify CAD vs. controls, predict major adverse cardiovascular events (MACE), arrhythmias, and post-infarction remodeling. Radiomics enabled differentiation of acute vs. chronic infarction and prediction of microvascular obstruction, sometimes from non-contrast CMR. Segmentation achieved high performance for myocardium (DSC up to 0.95), but scar and edema delineation were more challenging. Reported performance was moderate-to-high across tasks (classification AUC = 0.66–1.00; segmentation DSC = 0.43–0.97; radiomics AUC = 0.57–0.99). Despite promising results, limitations included small or overlapping datasets. In conclusion, AI and radiomics offer substantial potential to support diagnosis and prognosis of CAD through advanced CMR image analysis.

## 1. Introduction

Coronary artery disease (CAD) is a pathological process characterized by atherosclerotic plaque accumulation in the epicardial arteries, whether obstructive or non-obstructive, and represents a range of clinical syndromes caused by insufficient coronary blood flow to the myocardium [1,2]. Angiography is the gold standard for characterizing CAD; however, angiography is invasive and patients are exposed to high radiation and contrast doses. Cardiac computed tomography angiogram (CTA) is the most commonly used method for the evaluation of the coronary arteries in children [3,4] and adults, particularly in patients with low to moderate risk of CAD [5]; however, patients are exposed to high doses of radiation and iodinated contrast agents. Cardiovascular magnetic resonance (CMR) imaging has gained widespread acceptance for its non-invasive global assessment, including evaluation of cardiac anatomy and function, and the coronary arteries, the characterization of tissue, and the quantification of flow [6,7,8,9]. A valuable tool for the cardiovascular diagnosis [10,11] and prognosis in patients with CAD, CMR imaging can be used to measure markers of cardiac structure and function, and myocardial perfusion and scarring, and to provide detailed insights about myocardial tissue [12]. CMR imaging findings have been shown to have prognostic values. In particular, left ventricular ejection fraction (LVEF) has been shown to be an independent predictor of future cardiovascular events in patients with a recent myocardial infarction (MI) and RWMA, inducible perfusion defects, and LVEF have been shown to identify patients with suspected or known CAD [13,14]. Recently, radiomics has become a promising method for medical imaging and for the extraction and analysis of quantitative metrics from medical images, the so-called radiomic features that can be used to convert images into mineable data [15,16]. Radiomics makes it possible to characterize information that is imperceptible to human vision and impossible to analyse using traditional imaging techniques [15]. Radiomics might allow the identification of new non-invasive imaging biomarkers that could be useful for predicting the prognosis and response to treatment in patients with CAD [15].

Traditional CMR image analysis methods have also evolved to include the use of artificial intelligence (AI). For example, through machine learning (ML) and deep learning (DL) [17,18]. AI enables the creation of models and automatic assessment of cardiovascular segmentation, which improve the reproducibility and accuracy of the analysis compared with the performance of expert radiologists and cardiologists [19]. Radiomics and AI are closely interconnected through the ability of AI to manage and analyse the huge amount of data extracted from medical images compared with traditional statistical methods [20]. This comprehensive review explores recent applications of AI that are gaining traction in the field of CMR, as well as the use of radiomics in patients with CAD. These tools offer varied approaches to CMR image analysis, which requires a synthesis of the different approaches and their purposes, identifying their potential applications, benefits, limitations, and knowledge gaps in the literature from the last 10 years. The aim of this review was to identify whether and how these methods have been implemented in clinical practice and whether they have transformative potential in this field.

## 2. Materials and Methods

The present systematic review follows the Preferred Reporting Items for Systematic reviews and Meta-Analyses (PRISMA) guidelines [21]. However, this systematic review was not registered in any public registry of reviews and a protocol was not prepared. The literature from the last 10 years was identified from the MEDLINE, Web of Science, and Scopus databases up to 17 March 2025. The search strategy used the following terms: “coronary artery disease”, “ischemic heart disease”, “myocardial infarction”, “MRI”, “magnetic resonance imaging”, “CMR”, “cardiac magnetic resonance”, “artificial intelligence”, “machine learning”, and “deep learning”. These terms of interest were used with Boolean operators (Table 1).

All articles recovered from the systematic search were exported to the Rayyan web app [22], where duplicates were located and removed. The inclusion criteria used were as follows: original articles, studies based on humans, CAD population, AI, and English or Spanish language. Studies were excluded for the following reasons: duplicate studies, animal models, unpublished articles, grey literature, student dissertations or theses, book chapters, encyclopedias, or conference papers. Two independent reviewers (JS and MJ) performed two rounds of study selection: title and abstract screening followed by full-text review. Each article was screened by two independent reviewers, and disagreements were discussed with a third reviewer (RD) until consensus was achieved.

The authors extracted data that included general study information (author/s, study design, year of publication, country); clinical details (patient characteristics, age in years, sex, number of patients); AI models (ML and DL); targets; image acquisition details; and performance metrics according to the purpose of the study. The data extracted from the articles are presented in tables and analyzed by the authors. The quality of the included studies was assessed using the 44 verification criteria included in the Checklist for AI in Medical Imaging (CLAIM) [23].

## 3. Results

**Figure 1:**
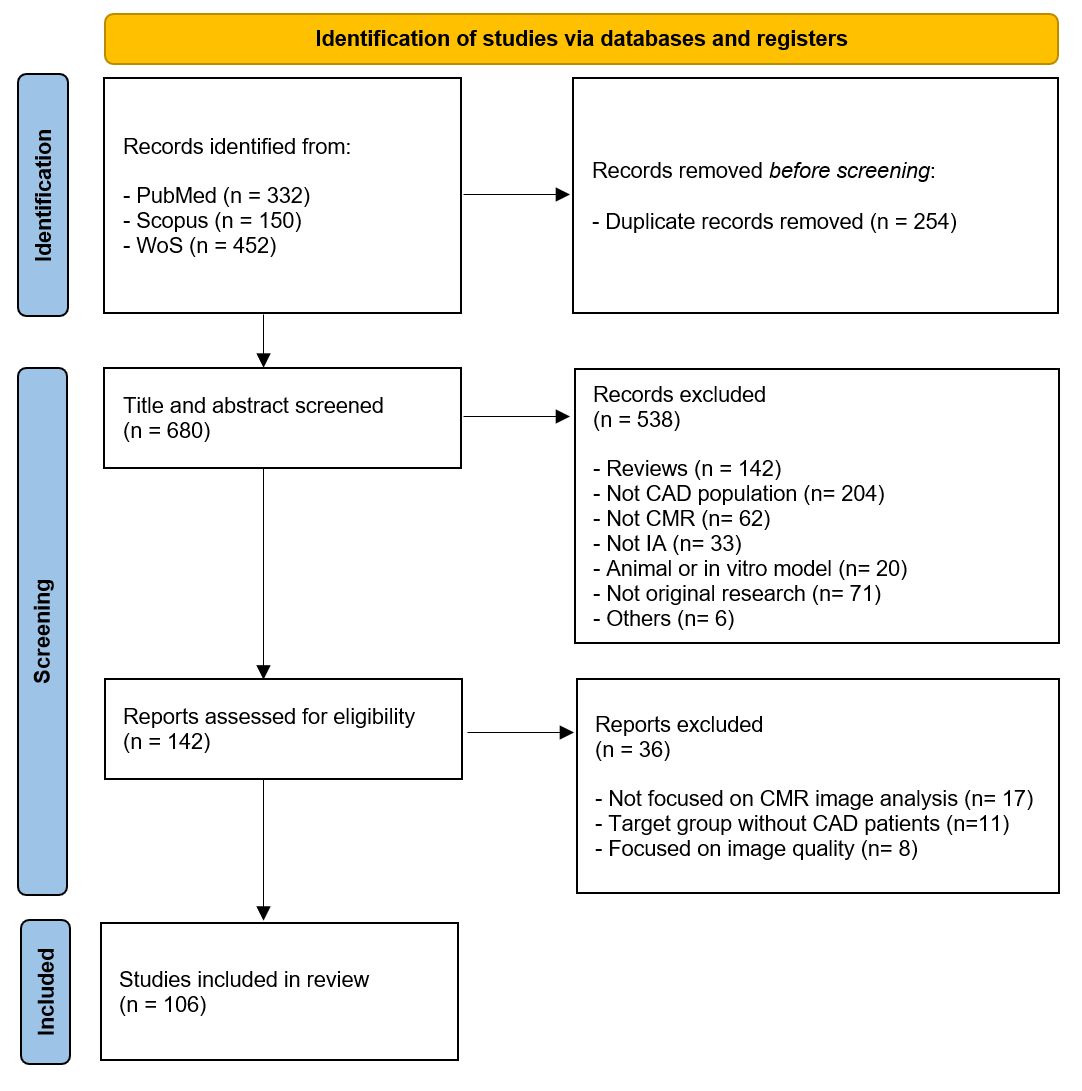
Flow diagram of study inclusion and exclusion criteria for this systematic review.

A total of 934 articles were found in the databases. After screening the titles and abstracts, 142 studies were considered relevant. Subsequently, a full-text assessment identified 106 studies that met the eligibility criteria (Figure 1). The studies showed a growing interest in the use of AI through ML and DL models over the past 10 years in the analysis of CMR images in CAD patients, with good diagnostic accuracy in the classification of CAD patients, as well as in the prediction of outcomes (CAD, arrhythmia, major adverse cardiovascular events “MACE”, or death) (n = 46); the segmentation of cardiac structures (myocardium and myocardial infarct, scar, or edema) (n = 41); and the application of radiomics for texture analysis (n = 19). The following sub-sections describe the findings of the studies analysed.

### 3.1. Classification

**Figure 2:**
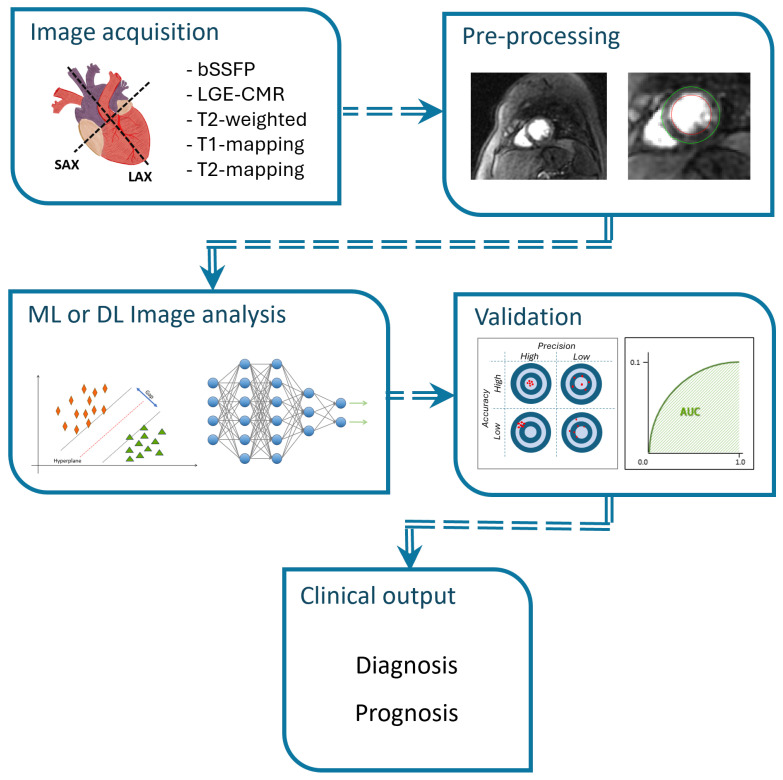
Classification steps for AI applications in CMR. (1) Acquisition of CMR images and clinical data. (2) Preprocessing of images through normalization, re-sizing for uniformity and data augmentation. (3) The data are split into training, validation, and test sets (e.g., 70/15/15). ML or DL models are trained on the training set for data analysis, and their performance is monitored using the vali-dation set. (4) The model is evaluated using the test set by calculating metrics such as accuracy (ACC) or area under the curve (AUC). (5) The output obtained is intended for diagnostic or prognostic purposes in CAD patients.

AI is attractive for clinical timescales wherein decisions must be made quickly on readily available computer systems. AI algorithms can learn outcomes based on key patient biomarkers using, for example traditional, ML techniques or even more sophisticated techniques such as DL/neural networks if distinguishing biomarkers are unknown, and these often provide superior accuracy and recall [24]. The steps used when applying AI algorithms of classification in CMR images are shown in Figure 2.

The characteristics of the forty-six included articles are provided in Table 2. The number of patients included in each study showed a high variability (range, 21–63,151). These articles included healthy volunteers and CAD patients, and the data came from both public and private databases. Thirty-four articles reported the number of subjects classified according to sex [25,26,27,28,29,30,31,32,33,34,35,36,37,38,39,40,41,42,43,44,45,46,47,48,49,50,51,52,53,54,55,56,57,58] and included a total of 35,916, men (65%) and 19,555 women (35%). Twenty-seven studies were conducted retrospectively [25,26,28,29,32,34,35,36,37,38,40,41,43,44,45,46,47,49,50,51,58,59,60,61,62,63,64] and eight prospectively [27,31,42,48,53,54,55,57]. Twelve were conducted across multiple centres, and 29 were performed at a single center. However, 15 articles did not provide a description of the databases used and the identification of aspects such as age, comorbidities, and clinical characteristics [24,33,51,52,59,60,61,62,63,64,65,66,67,68,69].

#### 3.1.1. Classification Between Healthy Volunteers and Patients with Cardiovascular Diseases

In the period analysed, 15 studies focused on distinguishing between, healthy volunteers, CAD patients, and patients with other cardiac pathologies [29,32,38,40,44,49,55,56,58,60,63,68,69,70]. Joloudari et al. [63] and Iqbal et al. [68], used of DL and ML applications, respectively, and reported high accuracy (ACC = 99.91% and 99.35%, respectively) in distinguising healthy volunteers from CAD patients. However, the study by Iqbal et al. [68], reported a lower ACC and included 63151 subjects compared with Joloudari et al. [63] who included only 30. Neither of these two studies provided a description of the study subjects; both mentioned only the number of patients and healthy subjects without describing other characteristics that could be relevant to this pathology, such as age, gender, or comorbidities. The study conducted by Paciorek et al. [44], compared the classification of the normal and abnormal myocardium from late gadolinium enhancement (LGE) and T1-mapping images, and reported a higher ACC for LGE images (ACC = 88%) than for T1-mapping (ACC = 70%).

#### 3.1.2. Risk Stratification for Major Adverse Cardiovascular Events (MACE)

Four articles focused on the assessment of the risk of CAD patients developing MACE [27,33,37,39,46,47,50], which provides very useful prognostic information for the management of CAD patients. The study by Knott et al. [39] used cvi42 commercial software (Circle Cardiovascular Imaging, Calgary, Alberta, Canada), and the study by Backhaus et al. [27] used suiteHEART com-mercial software (v4.0.6; Neosoft, Pewaukee, WI, USA). Schuster et al. [50] used both software programs for their analysis. Both software programs perform the analysis fully automatically. These studies demonstrate the utility of different AI software programs in predicting MACE in CAD patients (see Table 2).

#### 3.1.3. Risk Stratification for Arrhythmia-Induced Mortality in CAD Patients

Of the investigations targeting the prediction of arrhythmia and mortality in CAD pa-tients [24,25,26,36,43,45,48,57], the study by Maleckar et al. [24] achieved the best performance with an ACC of 86%, based on a database of 30 patients by combining patient data and computational simulation-supported data augmentation using an ML model. By contrast, although the study by Pezel et al. [45], did not report superior performance (AUC = 0.75), it included clinical and stress CMR data for 31,762 patients and found a higher prognostic value for predicting death compared with all traditional clinical or CMR scores.

#### 3.1.4. Early Identification of Left Ventricular Remodelling (LVR)

Left ventricular remodelling (LVR) is a risk in CAD [42,61]. Early detection of patients likely to undergo LVR can help to optimize therapeutic strategies aimed at preventing or reversing this condition and thereby reducing its subsequent clinical consequences [61]. Two studies using ML models aimed at predicting postinfarction LVR [42,61]. Although the study by Mauger et al. [42] included patients 10 or more years after the incident and the study by Dieu et al in 2022 was conducted 3 months after an MI both achieved similar performance values (AUC of 0.77 and 0.8, respectively).

#### 3.1.5. Identification of Normal and Infarcted Myocardial Segments

An important factor when analysing CMR images of CAD patients is the differentiation of infarcted and non-infarcted remote myocardial segments (using the 16-segment American Heart Association (AHA) nomenclature [71]. Some research groups have aimed to identify infarcted segments in the left ventricular (LV) myocardium [31,35,41,52,64,66,67]. Chen et al. [31] reported the highest ACC (87.6%) in 73 patients using a DL model that combines a stack denoising autoencoder (SDAE) with a support vector machine (SVM). Wang et al. [52] found an ACC of 86%, using a DL model (CNN ResNet50-AC) in 301 patients. Li et al. [41] classified segments as viable, remote, or non-viable based on myocardial transmural extension.

LGE is the type of CMR imaging used most often in CAD patients because it provides better contrast between healthy and diseased. However, there some patients have contraindications for gadolinium-based contrast injection, and some authors have proposed using other types of CMR imaging, such as balanced steady state free precession (bSSFP), to detect MI [69] (ACC = 86.39%) or MACE [27] (AUC = 0.69 for automatic analysis of global longitudinal strain (GLS) and AUC = 0.66 for automatic analysis of global circumferential strain (GCS). Aiming to improve efficiency, other studies, such as those conducted by Iqbal et al. [68] and Chen et al. [30], have used models that integrate the analysis of different types of images to classify patients (see Table 2).

#### 3.1.6. Other Classifications Findings

Other less-studied applications of CMR for CAD patients have been to identify LV paradoxical pulsation, such us in the study by Chen et al. [30], which achieved ACC values of 85% for the internal testing cohort and 84% for the external testing cohort. Goldfarb et al. [62] determined the viability and performance of water-fat separation by CMR imaging and parametric mapping using DL and found good correlations with the conventional model. Wu et al. [53] examined a compressed sensing AI framework to accelerate image acquisition in contrast-free wholeheart coronary MR bSSFP angiography and reported an ACC of 90% per patient. In the study by Chen et al. [65], the objective was to evaluate the myocardial protection of ivabradine (IBD) combined with trimetazidine (TMZ) in patients with CAD. For this purpose, Patients were randomized to groups A (treatment with TMZ), B (treatment with IBD), and C (combined treatment of IBD + TMZ), with 40 patients in each group. Following the study, the CNN algorithm obtained an accuracy of 91.04 and an AUC of 0.96 for the diagnosis of myocardial damage.

### 3.2. Classification Using Radiomics

**Figure 3:**
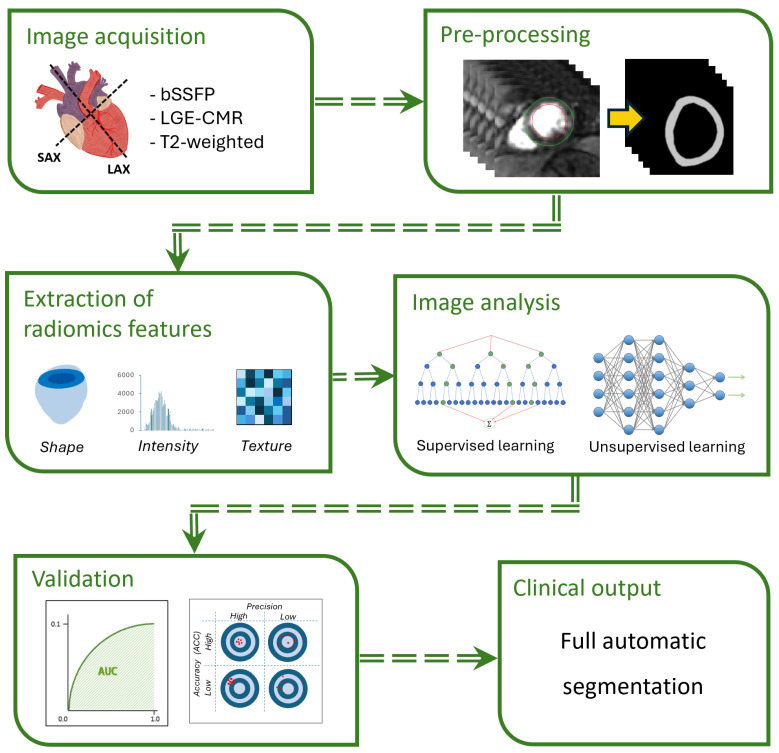
Radiomics features classification steps for AI CMR applications. (1) Acquisition of CMR images and clinical data. (2) Preprocessing of images through normalization, image resizing, and segmentation of the structures of interest (e.g., LV myocardium). (3) Features are extracted from the images, such as pixel intensity, to calculate homogeneity and contrast, shape of the segmented structures, and transformation features to capture more complex patterns (using transformations such as wavelet or Fourier). (4) Feature selection is performed by applying techniques such as PCA to reduce dimensionality or decision trees (DT) to identify the most relevant features. (5) The data are divided into training, validation, and test sets (e.g., 70/15/15). ML or DL models are trained for data analysis on the training set, and their performance is monitored using the validation set. (6) The model is evaluated using the test set by calculating performance metrics such as the AUC. (7) The output obtained comprises of the fully automated analysis of cardiac structures in CAD patients.

Radiomics is a quantitative image analysis method that can be used to extract highly detailed information about ventricular shape and myocardial characteristics and thereby provides new information from existing standard-of-care images [72]. Radiomics and AI are closely linked given the ability of AI to handle and analyse the large volumes of data extracted from medical images, which surpasses traditional statistical methods [20]. Radiomics features classification steps for AI CMR applications are shown in Figure 3. In Table 3, we describe the studies that used radiomics in the analysis of CMR. In these studies, the cohort size ranged from 43 to 63,648 and included healthy volunteers and patients, from public and private databases [73,74,75,76,77,78,79,80,81,82,83,84,85,86,87,88,89,90,91]. However, the number of men and women included were reported in only 12 studies [74,76,78,79,80,83,84,85,86,87,88,89] and represent a total of 1.410 men (75%) and 497 women (25%). Only one study was multicentric [89], 17 from a single medical centre [73,74,75,76,77,78,79,80,81,82,83,84,85,86,87,90,91], and one study did not report the location [90]. Twelve studies were conducted retrospectively [73,75,76,78,79,80,83,84,85,86,87,91] and four prospectively [74,81,88,90]. Seven studies did not report information about the database used [73,75,77,81,82,89,91], possibly because they used third-party databases.

The focus of the studies analysed varied. Three studies evaluated the ability of machine learning algorithms to use radiomic features extracted from cardiac magnetic resonance imaging (CMR) sequences for the differentiation of CAD patients [77,78,91]. Wang had the best ACC level (0.93), using the T1 + sBTFE imaging sequence. Six studies evaluated the extent of myocardial damage in CAD patients by identifying viable, non-viable, and remote tissue [73,74,75,80,84,86]. Ma et al. [86] evaluated the feasibility of texture analysis of non-contrast-enhanced T1 maps of CMR imaging for the diagnosis of myocardial injury in acute MI. They predicted various characteristics for the images, such as the presence of LGE, positive MVO segments, or non-irreversible injury at 6 months after MI and found the best results for identifying MVO when combining radiomics signatures and T1 values (AUC = 0.86). Larroza et al. [84] compared the accuracy of texture analysis on non-contrast MRI images with LGE MRI in a database of 50 subjects and obtained an AUC of 0.849, with showed that non-viable segments can be detected on cine MRI using texture analysis. However, another study by Abdulkareem et al. [73], which used a larger database of 272 subjects, yielded less promising results such as AUC values of 0.58 for SVM classification and 0.57 for decision tree (DT), which highlights the need for more robust studies on this topic.

The study by Durmaz et al. [79] was the only study aimed at predicting MACE in CAD patients using texture analysis combined with clinical variables and using various classification models, such as kNN, AdaBoost, random forest, SVM, naive Bayes, SGD, and neural networks (NN). They achieved the best results with NN (AUC = 0.965 and ACC = 0.894). Larroza et al. [83] and Baessler et al. [76] used radiomics to differentiate chronic and acute MI. The former compared the classification performance between LGE CMR images (AUC = 0.86) and cine CMR (AUC = 0.82). Baessler et al. [76] used only non-enhanced cine CMR and reported with very good results (AUC = 0.92). By contrast, Rauseo et al. [90], focused on defining the radiomic signatures that indicate the relationship between ischaemia processes occurring in the brain and heart. Radiomic signatures provided significantly better disease discrimination than conventional indices, as suggested by the AUCs: ischaemic heart disease: 0.82 vs. 0.75; cerebrovascular disease: 0.79 vs. 0.77; MI: 0.87 vs. 0.79; and ischemic stroke: 0.81 vs. 0.72.

### 3.3. Segmentation

**Figure 4:**
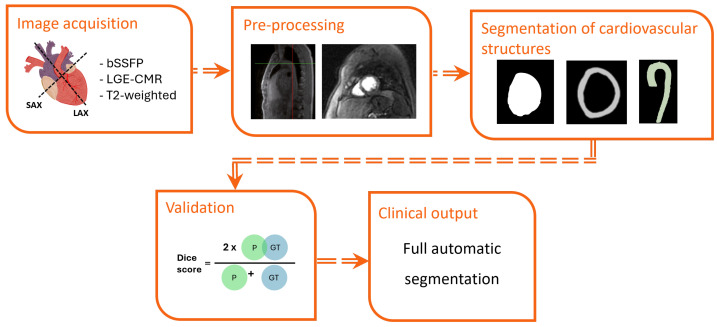
Segmentation steps for AI CMR applications. (1) Acquisition of CMR images. (2) Preprocessing of images through normalization, image resizing, and data augmentation. (3) The data are divided into training, validation, and test sets (e.g., 70/20/10). DL segmentation models (e.g., U-Net or SegNet) are trained for data analysis on the training set, and their performance is monitored using the validation set. (4) The model is evaluated using the test set by calculating segmentation-specific metrics such as the Dice score. (5) The output obtained comprises of the automatic segmentation of cardiac structures.

Segmentation steps for AI CMR applications are shown in Figure 4. A total of 41 articles reported the segmentation of different cardiac structures such as the myocardium, myocardial infarction scar (MIS), myocardial edema (ME), coronary artery or adipose tissue on CMR images of CAD patients (see Table 4). The size of the cohort used in these studies varied widely from 20 to 1354, and included both patients and healthy volunteers from private and public databases. Sixteen articles reported the number of men and women included in the studies [30,92,93,94,95,96,97,98,99,100]: a total of 1986 men (75%) and 679 women (25%). Eighteen studies were retrospective [92,93,94,95,96,98,99,101,102,103,104,105,106,107,108,109,110,111], and only one mentioned being prospective [112]. Fifteen studies were multicentre [92,95,97,100,105,107,108,109,111,112,113,114,115,116,117], thirteen obtained data from a single medical centre [70,94,96,98,99,102,103,104,110,118,119,120,121], and 13 articles did not provide a description of the databases used.

The AI models were used for the segmentation of different cardiovascular structures. Generally, these studies used of Dice similarity coefficient (DSC) as an evaluation metric to evaluate the similarity between a predicted segmentation mask and the ground truth segmentation mask [122]. The DSC ranged from 0, indicating no overlap, to 1, indicating perfect overlap. Fourteen of the studies evaluated myocardium segmentation in CAD patients and healthy controls compared with the ground truth [92,95,99,102,103,106,111,113,115,118,119,121,123,124]. Yan et al. [121] studied a cohort of 1354 subjects and achieved the best results for myocardial segmentation with DSC values of 0.94 for training, 0.87 for validation, and 0.94 for testing. Ten studies segmented the Myo as well as the MI or MIS [70,96,97,98,100,107,108,109,110,112,120,125]. The studies by Lecesne et al. [125], and Heidenreich et al. [96] presented their segmentation accuracy results for Myo and MI or MIS separately. Lecesne et al reported better results with a DSC of 0.92 for Myo and 0.92 for MI for a database of 150 subjects. Other studies focused on the segmentation of MI or MIS. Zabihollahy et al. [100] obtained a DSC of 0.93 from an LGE MRI database of 34 subjects. This finding was similar to that of Xu et al. [109] (DSC of 0.65), who used a database for 165 subjects. The study by Xu et al. [109] was notable for using cine CMR images with no contrast, which is useful for patients for whom contrast agents cannot be administered [126].

Other studies have focused on segmenting MI and ME [104,116,126,127,128,129]. These studies performed segmentation using multisequence images, including bSSFP, LGE, T2-weighted, and T1- and T2-mapping images, and aimed to leverage the characteristics of each type of image. For example, LGE provides information about the infarcted region, T2 weighting allows the visualization of ME, and bSSFP CMR provides information about anatomical structural [129], and global or RWMA, which are complementary sequences when an-alysing CMR data. Only one study focused on automating coronary artery segmentation for CAD diagnosis [105].

Studies of segmentation of myocardial infarction and microvascular obstruction (MVO) have been conducted by Brahim et al. [93], de la Rosa et al. [130], and Brahim et al. [131]. These authors used LGE CMR and DL models, and Brahim et al. [93] achieved the best results with DSC values of 0.95 for Myo, 0.78 for MI, and 0.77 for MVO. They also classified the regions based on whether they corresponded to Myo, MI, or MVO, with an ACC of 98%.

Chen et al. [94] studied segmented pericardial adipose tissue, which is associated with different cardiovascular diseases, including CAD. They used a different evaluation metric than DSC, called the Hausdorff distance (HD), whose use is increasing in medical image segmentation [132]. Unlike earlier metrics that examine every pixel, HD considers only the boundary pixels of every patch of pixels belonging to the same class. It calculates the distance between every corresponding point within one or two boundaries [133]. Chen et al. [94] obtained an HD of 15.62 ± 18.61 mm using a database of 150 LGE MRI images and concluded that these results are only an indicator but have potential in diagnosing cardiovascular pathologies, including CAD.

**Table 4:** Segmentation studies in CMR image in CAD.

Reference	# Subject (M/F)	Age(y) Mean ± Std	CMR Seq.	AI Model	Target	Performance
Gröschel, et al. [95]	136 (91/45)	HS 44 ± 16 CAD 68 ± 11	LGE	Deep CNN	LV-Myo	Healthy: DSC = 0.85 Patients: DSC = 0.80
Mosquera- Rojas, et al. [106]	274 (NR)	NR	LGE	DualUNet	LV-Myo	DSC = 0.84
Barbaroux, et al. [92]	271 (197/73)	48 ± 14	LGE	DynU-Net	LV-Myo	SAx: DSC = 0.83 LAx: DSC = 0.82
Yan, et al. [121]	1354 (NR)	NR	LGE	SegNet model	LV-Myo	Train: DSC = 0.94 Validation: DSC = 0.87 Test: DSC = 0.94
Scannell, et al. [99]	175 (136/39)	64 ± 10	T1-w	U-Net	LV-Myo	DSC = 0.80
Ahmad, et al. [113]	56 (NR)	58	LGE	DL	LV-Myo	DSC = 0.85
Kim, et al. [115]	35 (NR)	NR	bSSFP	DL (CNN- U-Net)	LV-Myo	DSC = 0.80
Liu, et al. [123]	32 (NR)	NR	T2-w LGE	CLS	LV-Myo	DSC = 0.84 DSC = 0.78
Tan, et al. [124]	1340 (NR)	NR	bSSFP	CNN (three networks (LM, CTR, MB))	LV-Myo	DSC = 0.86
Chen, et al. [119]	150 (NR)	NR	T1-w LGE	Res-UNet	LV-ED LV-ES RV-ED RV-ES Myo ED Myo ES	DSC = 0.89 DSC = 0.81 DSC = 0.81 DSC = 0.70 DSC = 0.72 DSC = 0.76
Papetti, et al. [107]	144 (NR)	NR	LGE	CNN	LV-Myo MIS	DSC = 0.79 DSC = 0.78
Lecesne, et al. [125]	150 (NR)	NR	LGE	U-Net	LV-Myo MI	DSC = 0.92 DSC = 0.92
Lin, et al. [97]	34 (29/5)	NR	LGE	CTAEM-Net	MIS	DSC = 0.90
Mamalakis, et al. [120]	20 (NR)	NR	LGE	BZ- SOCRATIS	Myo Core Scar Border Scar Myo Core Scar Border Scar	Internal: DSC = 0.81 DSC = 0.60 DSC = 0.43 External: DSC = 0.70 DSC = 0.44 DSC = 0.54
Xu, et al. [110]	165 (NR)	NR	LGE	BMAnet	MI	Labeled 33: DSC = 0.59 Labeled 66: DSC = 0.65
Chen, et al. [70]	195 (NR)	NR	LGE	U-Net	MI	DSC = 0.84
Heidenreich, et al. [96]	78 (64/14)	64	LGE	nnU-nets	Myo MIS	DSC = 0.83 DSC = 0.72
Xu, et al. [109]	165 (NR)	NR	bSSFP	DSTGAN	MIS	DSC = 0.92
Zabihollahy, et al. [100]	34 (29/5)	51 ± 12	LGE	CNN-based	MIS	DSC = 0.93
Moccia, et al. [98]	30 (26/4)	NR	LGE	FCNN	MIS	DSC = 0.71
Li, et al. [134]	NR (NR)	NR	bSSFP LGE T2-w	NVTrans- UNet	MI MI+ME	DSC = 0.64 DSC = 0.57
Qiu, et al. [116]	NR (NR)	NR	Multi- sequence: bSSFP LGE T2-w T1-mapping T2-mapping	MyoPS-Net	MIS ME	DSC = 0.65 DSC = 0.74
Cui, et al. [128]	45 (NR)	NR	LGE T2-w bSSFP	U-Net++ (Deep supervision) + EfficientSeg- B1 (Ours)	MIS MIS + ME Average	DSC = 0.71 DSC = 0.74 DSC = 0.72
Li, et al. [129]	45 (NR)	NR	LGE T2-w bSSFP	TAUNet	LV RV Myo MIS ME	DSC = 0.94 DSC = 0.91 DSC = 0.91 DSC = 0.62 DSC = 0.78
Cui, et al. [127]	45 (NR)	NR	LGE T2-w bSSFP	Deep U-net Deep U-net+ DFM	MIS MIS+ME MIS MIS+ME	DSC = 0.68 DSC = 0.70 DSC = 0.69 DSC = 0.70
Brahim, et al. [93]	150 (89/61)	NR	LGE	ICPIU-Net	Myo MI MVO Classification	DSC = 0.95 DSC = 0.78 DSC = 0.77 ACC = 98,00
de la Rosa, et al. [130]	100 (NR)	NR	LGE	CNN	MI+MVO	DSC = 0.77
Brahim, et al. [131]	150 (NR)	NR	LGE	3D pretrained Autoencoder network and the 3D U-Net	Myo MI MVO	DSC = 0.95 DSC = 0.76 DSC = 0.73
Chen, et al. [94]	150 (92/58)	HS 32 ± 12 CAD 61 ± 12	LGE	3SUnet	Cardiac adipose tissue	HF = 15.62
Arega, et al. [114]	295 (NR)	NR	T1-mapping (Native) T1-mapping (Post- Contrast) T1-mapping (Native) T1-mapping (Post- Contrast)	Swin-based U-Net CNN-based U-Net	LV MYO RV LV MYO RV LV MYO RV LV MYO RV	DSC = 0.97 DSC = 0.91 DSC = 0.92 DSC = 0.95 DSC = 0.88 DSC = 0.89 DSC = 0.96 DSC = 0.90 DSC = 0.90 DSC = 0.94 DSC = 0.85 DSC = 0.86
Popescu, et al. [135]	401 (NR)	NR	LGE bSSFP	DNN	LV Myo MIS	DSC = 0.93 DSC = 0.57
Mamalakis, et al [136]	60 (NR)	NR	LGE	MA- SOCRATIS	LV Myo MIS	intra-observer: DSC = 0.81 inter-observer: DSC = 0.70 intra-observer: DSC = 0.70 inter-observer: DSC = 0.70
Al-antari, et al. [101]	150 (89/61)	NR	LGE	ResU-Net	MI+MVO	ACC = 88.50
Jani, et al. [112]	501 (431/70)	59 ± 12	LGE	Cascaded U-Net	LV-Myo MIS	DSC = 0.66 DSC = 0.75
Qi, et al. [108]	415 (370/45)	59 ± 10	CGE	DGL	MIS	ACC = 0.92
Yalcinkaya, et al. [111]	150 (NR)	60 ± 14	LGE bSSFP	DNN	LV-Myo	Internal: DSC = 0.89 External: DSC = 0.88
Lin, et al. [105]	174 (119/55)	51 ± 12	Cine	U-Net	Coronary artery	Training: DSC = 0.95 Validation: DSC = 0.94
Ben Khalifa, et al. [103]	163 (40/123)	HS 42 ± 14 CAD 58 ± 11	LGE bSSFP	U-Net	LV-Myo Classification MI	DSC = 0.92 ACC = 0.96
Li, et al. [104]	45 (NR)	NR	LGE T2-w bSSFP	DNN (MPS- Mamba)	MIS ME	DSC = 0.71 DSC = 0.73
Bernardo, et al. [118]	171 (NR)	NR	NR	U-Net	LV Myo	ED: DSC = 0.94 ES: DSC = 0.79 ED: DSC = 0.81 ES: DSC = 0.69
Jafari, et al. [102]	55 (37/18)	50 ± 17	DCE	U-Net	LV-Myo	DSC = 0.78

# = number; MI = myocardial infarction; HS = Healthy Subject; MIS = myocardial infarction scar; ME = myocardial edema; Myo = myocardium; MVO = microvascular obstruction; bSSFP = balanced steady-state free precession; DL = deep learning; SVM = Support vector machine; LGE = late gadolinium enhancement; DCE = dynamic contrast enhanced; CMR = cardiac magnetic resonance; LV = left ventricle; RV = right ventricle; ED = end-diastolic; ES = end-systolic; ACC = accuracy; DSC = dice similarity coefficient; CNN = convolutional neural network; DNN = deep neural network; CLS = coupled level set; LM = landmarks network; DGL = Deep Generative Learning; CTR = center point network; CTAEM-Net = cascaded triplanar autoencoder M-Net; MB = myocardial boundaries network; FCNN = fully convolutional neural network; DSTGAN = deep spatio-temporal adversarial network; NR = Not reported.

### 3.4. Quality Assessment

Assessment of the studies using the Checklist for AI in Medical Images (CLAIM) [23] showed an overall compliance of 81%. However, many studies lacked a description of de-identification methods for clinical trial registration and explainability or interpretability methods (see Supplementary Material, CLAIM Quality).

## 4. Discussion

This analysis further supports the consolidated position of CMR as a non-invasive reference modality for the diagnostic and prognostic assessment of coronary artery disease, and how the use of AI and radiomics improves diagnostic accuracy and efficiency in clinical workflow.

In the classification between healthy volunteers and patients with cardiovascular diseases, one of the most developed areas has been the binary classification between healthy individuals and patients with CAD. Although some studies, such as those by Joloudari et al. [63] and Iqbal et al. [68], report extremely high accuracy values (>99%), their clinical validity is questionable due to the lack of transparency in population characterization. This absence of information may conceal biases in data collection or class composition. Moreover, other studies, such as that by Paciorek et al. [44], show that the choice of imaging sequence (LGE vs. T1-mapping) significantly influences model performance, highlighting the importance of selecting the appropriate imaging modality.

Studies aimed at predicting MACE and arrhythmias in CAD patients have demonstrated the potential clinical value of AI in personalised medicine. However, the investigations with the highest reported accuracy (e.g., Maleckar et al. [24]) often rely on small databases and artificial data augmentation, which limits their generalisability to real-world clinical settings. In contrast, studies such as that by Pezel et al. [45], though reporting lower performance (AUC = 0.75), are based on large clinical cohorts (>30,000 patients), thereby offering more robust and clinically relevant conclusions.

The prediction of post-infarction left ventricular remodelling represents a clinically relevant but still underexplored application. The few available studies, such as those by Mauger et al. [42] and Dieu et al. [61], show consistent performance (AUC = 0.78), yet they differ considerably in the time post-infarction at which patients were analysed (months vs. decades), suggesting the need for more longitudinal research and standardised cohort timelines.

Some innovative applications—such as the detection of left ventricular paradoxical pulsation, contrast-free imaging analysis, or the evaluation of combined pharmacological therapies—illustrate the potential of AI in less explored yet clinically meaningful areas. Although preliminary, these studies open up new research avenues, especially relevant in contexts where conventional methods present limitations in terms of time, cost, or accessibility.

The integration of radiomics has demonstrated its ability to identify quantitative information imperceptible to the human eye, even in non-contrast images. Some studies have shown its usefulness in distinguishing between acute and chronic infarction, predicting microvascular obstruction (MVO), and stratifying the risk of MACE.

Only three studies specifically investigated the use of radiomics features to differentiate CAD patients from healthy individuals. Among these, the work by Wang et al. [137] achieved the highest accuracy (ACC = 0.93) using a T1 + sBTFE imaging sequence, demonstrating the potential of radiomics in disease classification. Nevertheless, the small number of studies focused on this task calls for further research to validate and extend these findings.

Several other studies addressed the classification of myocardial tissue status—viable, non-viable, or remote—particularly in the context of infarction. These studies varied widely in their imaging sequences and computational approaches. For example, Ma et al. [86] demonstrated that combining radiomics signatures with T1 mapping yielded promising results for predicting microvascular obstruction (AUC = 0.86). Larroza et al. [84] also reported a high AUC (0.84) for detecting non-viable segments using cine MRI. However, the study by Abdulkareem et al. [73], which used a larger cohort, yielded substantially lower performance (AUC = 0.58), illustrating the current inconsistency and underscoring the need for more robust, standardised protocols and larger, better-characterised cohorts.

Only one study, conducted by Durmaz et al. [79], examined the use of radiomics for predicting major adverse cardiovascular events (MACE). Their findings, particularly the superior performance of neural networks (AUC = 0.96, ACC = 89.4), suggest that combining radiomic features with clinical data may significantly enhance predictive performance. This integrative approach could represent a future direction for risk stratification models.

Radiomics has also been used to differentiate between acute and chronic myocardial infarction. Larroza et al. [83] and Baessler et al. [76] demonstrated that both LGE and cine CMR sequences can be effective in this differentiation, with Baessler et. al achieving particularly high performance using only non-enhanced images (AUC = 0.92). These findings indicate that radiomics may offer alternatives to contrast-enhanced imaging in patients with contraindications to gadolinium administration.However, the accuracy and robustness of radiomics must be validated in larger, external, and multicenter cohorts, using standardized scanning parameters, to fully establish its reliability and added value in clinical practice.

Most studies evaluated segmentation performance using the Dice Similarity Coefficient (DSC), a standard metric that quantifies the spatial overlap between predicted and ground truth masks. Among the studies focused on myocardium segmentation, Yan et al. [121] achieved some of the highest DSC scores reported (up to 0.94 in both training and testing sets), which reflects strong agreement with manual annotations. Other studies, such as those by Lecesne et al. [125] and Zabihollahy et al. [100], also reported high DSC values for both Myo and infarcted regions, suggesting that AI models can perform comparably to human experts in segmenting key cardiac structures.

However, there is noticeable variability in segmentation accuracy across studies, which may relate to differences in imaging sequences (e.g., cine vs. LGE), dataset size, and model architecture. For example, Xu et al. [56] reported significantly lower DSCs (0.652) when using cine CMR without contrast, which highlights the challenges of achieving precise infarct segmentation in contrast-free protocols a relevant issue in patients contraindicated for gadolinium administration.

The quality assessment of the included studies, conducted using the Checklist for Artificial Intelligence in Medical Imaging (CLAIM) [23], revealed an overall compliance rate of 81%, suggesting that most studies adhered to key reporting standards. However, notable deficiencies were identified, particularly regarding the reporting of data de-identification procedures, clinical trial registration, and the inclusion of explainability or interpretability methods. These omissions are concerning given the increasing emphasis on transparency, reproducibility, and ethical use of AI in clinical practice. The limited attention to interpretability also raises questions about the clinical trustworthiness and usability of the models, especially in high-stakes decision-making scenarios. Addressing these gaps is essential to enhance the credibility and translational potential of AI applications in cardiac imaging.

On the other hand, of the 106 studies analysed, 49.1% were conducted in Europe, 24.5% in Asia—of which 69.2% were carried out in China—and 8.5% in North America. In contrast, Africa and Latin America were markedly underrepresented, accounting for only 2.8% and 0.9% of the studies, respectively. These findings highlight the need to promote research in underrepresented regions to generate contextually relevant evidence for these populations.

Furthermore, the studies analysed had several limitations. One of the main ones is the sample size. There is a repetitive use of similar databases, single-center studies, a lack of external validation, and a lack of prospective studies. Additionally, the imbalance between groups and the lack of population diversity make it difficult to generalize the results. This is reflected in the gender disparity, with a marked underrepresentation of female participants. Only three studies included a majority female population; however, these women were not part of the groups diagnosed with coronary artery disease (CAD). Some studies identified the use of commercial software, which limits transparency in data processing. Overall, the methodological quality of the studies is acceptable, but there are areas that require strengthening to ensure greater transparency and reproducibility. Finally, the quality of the ground truth was limited because several studies relied on a single expert.

Despite following a rigorous methodology for conducting this systematic review, several limitations were identified that should be considered when interpreting the results. First, the literature search was restricted to publications in English and Spanish, which may have led to the exclusion of relevant studies published in other languages. Second, although multiple databases were consulted (such as PubMed, Scopus, and Web of Science), there is a potential for publication bias, as studies with negative or non-significant results are less likely to be published. Additionally, some potentially relevant studies may have been omitted due to lack of access to full texts or limitations in the search terms used.

Another limitation was the process of quality assessment and data extraction. Although this was performed independently by two reviewers, it may still be subject to inevitable subjective bias.

Therefore, it is necessary to move toward multicenter, prospective studies that integrate relevant clinical variables, which will allow us to consolidate the clinical impact of these emerging tools, such AI and radiomics, in cardiovascular precision medicine.

## 5. Conclusions

This systematic review provides a synthesis of the use of AI tools described in the literature for CMR image analysis in patients with CAD. Various purposes for using these tools were identified, such as: diagnostic and/or prognostic prediction, segmentation of anatomical structures, and quantification of hemodynamic parameters. Although the number of studies has increased between 2015 and 2025 and these have generally shown very good results. However, they have limitations, including the use of common data-bases, some using small cohorts, and some not describing the population by identifying demographic or clinical characteristics. Therefore, to translate these studies into clinical practice, it is important that future studies expand the dataset, include a greater diversity of controls and healthy patients, and incorporate relevant clinical and demographic information to improve ACCs. Radiomics combined with AI models has been shown to have potential for improving the diagnostic and prognostic accuracy of CAD patients, even in non-contrast CMR images, though improvements and more valiation studies are necessary for clinical implementation.

Finally, the use of these applications is gaining ground in the field of CMR, and, together with their integration in future studies, could have a significant impact on the diagnosis, prognosis and management of CAD patients, and thereby support specialists in their work in this field.

## Figures and Tables

**Table 1:** Detailed search strategy.

Database	Search Parameters
Pubmed	((((coronary artery disease) OR (ischemic heart disease)) OR (myocardial infarction)) AND ((((MRI) OR (Magnetic resonance imaging)) OR (CMR)) OR (Cardiac Magnetic resonance))) AND (((artificial intelligence) OR (machine learning)) OR (deep learning))
WOS	(((ALL = (coronary artery disease)) OR ALL = (ischemic heart disease)) OR ALL = (myocardial infarc-tion)) AND (((((ALL = (MRI)) OR ALL = (Magnetic resonance imaging))) OR ALL = (CMR)) OR ALL = (Cardiac Magnetic resonance)) AND (((ALL = (artificial intelligence)) OR ALL = (machine learn-ing)) OR ALL = (deep learning))
Scopus	(coronary AND artery AND disease OR ischemic AND heart AND disease OR myocardial AND infarction) AND (mri OR magnetic AND resonance AND imaging OR cmr OR cardiac AND mag-netic AND resonance) AND (artificial AND intelligence OR machine AND learning OR deep AND learning)

**Table 2:** Classification studies in CMR image in CAD.

Reference	# Subject (M/F)	Age(y) Mean ± Std	CMR Seq.	Target	(Category) AI Model	Performance
Bekheet, et al. [60]	1140 (NR)	NR	LGE	Myo Fibrosis +/−	(ML) MobileNetV2 GoogleNet ResNet50 FibrosisNet	ACC = 87.13 ACC = 88.60 ACC = 88.45 ACC = 96.05
Lalande, et al. [40]	150 (89/61)	MI 66 ± 14 HS 59 ± 12	LGE	MI +/−	(DL) Multi-input classification: CNN, RF.	ACC = 92.00 AUC = 0.96
Chen, et al. [32]	150 (89/61)	MI 66 ± 14 HS 59 ± 12	LGE	MI +/−	(ML) RF Regressor	Infarction: ACC = 88.67 PMVO: ACC = 77.33
Muthulakshmi, et al. [69]	21 (NR)	NR	bSSFP	MI +/−	(DL) Levenberg- Marquardt learning CNN	ACC = 86.39
Xu, et al. [56]	58 (36/22)	51 ± 16	LGE	MI +/−	(ML) SVM	ACC = 93.30
Attallah, et al [59]	100 (NR)	NR	LGE	MI +/−	(DL) Auto-MyIn	ACC = 98.40
Zhang, et al. [58]	299 (213/86)	HS 40 ± 13 CAD 56 ± 11	Non- contrast bSSFP	Chronic MI +/−	(DL) NR	AUC = 0.94
Joloudari, et al. [63]	30 (NR)	NR	NR	CAD +/−	(DL) FCM-DNN	ACC = 99.91 AUC = 1.00
Iqbal, et al. [68]	63151 (NR)	NR	LGE Perfusion T2w bSSFP	CAD +/−	(ML) LWNN (adapted version of LeNET5 model) NN	ACC = 99.35 AUC = 0.99
Wu, et al. [54]	64 (33/31)	59 ± 10	Non- contrast bSSFP	CAD +/−	(DL) CSAI	Patient: ACC = 87.50 Vessel: ACC = 91.10 Segment: ACC = 96.60
Chen [65]	120 (63/57)	Group A 64 ± 9 Group B 62 ± 8 Group C 62 ± 9	LGE	Myo injury	(DL) CNN	ACC = 91.04 AUC = 0.96
Paciorek, et al. [44]	200 (132/68)	53 ± 19	LGE T1-mapping	Normal/ Abnormal	(DL) DenseNet-161 (LGE PSIR) DenseNet-161 (T1 mapping)	ACC = 88.00 AUC = 0.75 ACC = 70.00 AUC = 0.69
Backhaus, et al. [27]	1095 (820/275)	64	bSSFP	MACE +/−	(DL) Commercial software	Auto-GLS: AUC = 0.69 Auto-GCS: AUC = 0.66
Schuster, et al. [50]	1017 (763/254)	64	LGE	MACE +/−	(DL) Commercial software	Auto-mated: AUC = 0.67 Auto corrected: AUC = 0.68
Pezel, et al. [46]	2152 (1653/499)	66 ± 12	LGE	MACE +/−	(ML) U-net Dijkstra’s algorithm	ICC = 0.83 (95% CI)
Knott, et al. [39]	1049 (702/347)	60 ± 13	Perfusion	Stress MBF and MPR associated with death or MACE	(NR) Commercial software	MBF: ICC = 0.68 (95%CI) MPR: ICC = 0.68 (95%CI)
Popescu, et al. [48]	269 (233/36)	61 ± 11	LGE	SCDA risk +/−	(DL) NN Architecture SSCAR	Internal: ACC = 77.00 External: ACC = 73.00
Pezel, et al. [45]	31762 (20,879/10,883)	63 ± 12	LGE	SCDA risk +/−	(ML) RSF	AUC = 0.75
Maleckar, et al. [24]	30 (NR)	NR	LGE	Arrhythmia risk +/−	(ML) NR	ACC = 86.00
Ghanbari, et al. [36]	761 (671/90)	65 ± 11	LGE	Arrhythmia risk +/−	(ML) Ternaus network (Multivariable Cox models– total scar) CNN	AUC = 0.67
Okada, et al. [43]	122 (106/16)	60 ± 11	LGE	Arrhythmia risk +/−	(ML) SVM+poly	ACC = 81.00
Zaidi, et al. [57]	397 (346/51)	64 ± 9	LGE	Major arrhythmic event +/−	(ML) Multivariate cox regression analysis	AUC = 0.81
Chen, et al. [30]	311 (294/17)	NR	T2w-STIR bSSFP T2-mapping LGE	Paradoxical pulsation +/−	(DL) CNN	Internal: ACC = 85.00 AUC = 0.91 External: ACC = 84.00 AUC = 0.83
Paciorek, et al. [44]	200 (132/68)	53 ± 19	LGE T1-mapping	Normal/ Abnormal	(DL) DenseNet-161 (LGE PSIR) DenseNet-161 (T1 mapping)	ACC = 88.00 AUC = 0.75 ACC = 70.00 AUC = 0.69
Chen, et al. [31]	73 (51/22)	NR	LGE	Segment Infarct +/−	(DL) SDAE+SVM	ACC = 87.60
Feng, et al. [66]	30 (NR)	NR	bSSFP LGE	Segment Infarct +/−	(ML) SVM-RFE	Basal: ACC = 80.50 Middle: ACC = 87.90 Apical: ACC = 81.00
Kim, et al. [64]	170 (NR)	NR	LGE	Segment Infarct +/−	(DL) ResNet50	ACC = 81.10 AUC = 0.87
Wang, et al. [52]	301 (172/129)	57	LGE	Segment Infarct +/−	(DL) MI-ResNet50- AC CNN	AUC = 0.86
Hernández- Casillas, et al. [67]	35 (NR)	NR	LGE	Segment Infarct +/−	(ML) Naïve Bayes	AUC = 0.69
Mauger, et al. [42]	5098 (2451/2565)	HS 60 ± 9 CAD 66 ± 9	GRE	Relationship between LV 3D shape CMR and incident cardio- vascular events	(ML) Model 3 (model 1+30 event-specific remodeling signatures derived from the PLS analysis)	AUC = 0.77
Dieu, et al. [61]	443 (NR)	NR	NR	LV remodeling +/−	(ML) LR	AUC = 0.78
Böttcher, et al. [28]	50 (37/13)	57	bSSFP	Myo function	(DL) Commercially available software	LV EDV: ICC = 0.99 LV ESV: ICC = 0.99 LV SV: ICC = 0.89 LV EF: ICC = 0.97 LV mass: ICC = 0.99
Goldfarb, et al. [62]	64 (NR)	NR	bSSFP	Water–Fat	(DL) U-Net	R2 ≥ 0.97 (*p* < 0.001)
Wu, et al. [53]	50 (15/35)	HS 24 ± 8 CAD 60 ± 12	Non- contrast bSSFP	Angiography	(DL) CSAI	Patient: ACC = 90.00 Vessel: ACC = 91.70 Segment: ACC = 97.30
Paciorek, et al. [44]	200 (132/68)	53 ± 19	LGE T1-mapping	Normal/ Abnormal	(DL) DenseNet-161 (LGE PSIR) DenseNet-161 (T1 mapping)	ACC = 88.00 AUC = 0.75 ACC = 70.00 AUC = 0.69
Cau, et al. [29]	107 (72/35)	61	bSSFP	CAD +/−	(ML) GB-GAM	AUC = 0.82
Alskaf, et al. [25]	1286 (845/441)	<65 65–75 >75	Perfusion	Mortality risk +/−	(ML) HNN	AUC = 0.82
Alskaf, et al. [26]	2740 (1726/1014)	<65 65–75 >75	LGE	Mortality risk +/− Arrhythmia risk +/−	(ML) HNN	Mortality: AUC = 0.77 Arrhythmia: AUC = 0.75
Corral-Acero, et al. [33]	1021 (NR)	63	LGE T1-w	MACE +/−	(DL) UNet	AUC = 0.77
Li, et al. [41]	42 (33/9)	60 71 ± 11	LGE bSSFP	Remote Viable Unviable	(ML) SVM XGBoost NN	Remote vs. Viable: AUC = 0.65 Viable vs. Unviable: AUC = 0.77 Remote vs. Unviable: AUC = 0.89
Udin, et al. [51]	279 (168/111)	HS 58 CAD 63	LGE	MI +/−	(ML) ResNet50 ResNet152V2	Without LWP: AUC = 0.76 With LWP: AUC = 0.88 Without LWP: AUC = 0.76 With LWP: AUC = 0.90
Frøysa, et al. [34]	41 (33/8)	58 ± 12	LGE	MI +/−	(ML) Texture-based probability mapping	R2 (*p* < 0.001)
Ghaffari- Jolfayi, et al. [35]	79 (52/27)	47 ± 12	LGE, T1 mapping T2 mapping	Segment Infarct +/−	(ML) RF	LAD territory: AUC = 0.89 RCA territory: AUC = 0.90 LCX territory: AUC = 0.92
Jacob, et al. [38]	1337 (602/735)	HS 50 ± 16 CAD 63 ± 12	bSSFP	CAD +/−	(DL) RF XGBoost	ACC = 0.81 AUC = 0.85
Righetti, et al. [49]	206 (164/42)	67	bSSFP	CAD +/−	(DL) U-Net	ACC = 79.00
Paciorek, et al. [44]	200 (132/68)	53 ± 19	LGE T1-mapping	Normal/ Abnormal	(DL) DenseNet-161 (LGE PSIR) DenseNet-161 (T1 mapping)	ACC = 88.00 AUC = 0.75 ACC = 70.00 AUC = 0.69
Wu, et al. [55]	99 (49/50)	HS 28 ± 11 CAD 59 ± 10	bSSFP	CAD +/−	(DL) DL-CS mDIXON	ACC = 84.10
Guglielmo, et al. [37]	730 (616/114)	63 ± 10	LGE	MACE +/−	(DL) FCN U-Net	HR = 1.08 (95% CI)
Pezel, et al. [47]	2038 (947/1091)	70 ± 12	LGE Perfusion	MACE +/−	(ML) XGBoost	Internal: AUC = 0.86 External: AUC = 0.84 AUC = 0.92

# = number; CAD = coronary artery disease; HS = healthy subjects; MI = myocardial infarction; bSSFP = balanced steady-state free precession; Myo = myocardium; ML = machine learning; DL = deep learning; SVM = support vector machine; poly = polynomial kernel; RFE = recursive feature elimination; NN = neural network; LR = logistic regression; RF = random forest; RSF = random survival forest; LWNN = light-weight neural network; LGE = late gadolinium enhancement; CMR = cardiac magnetic resonance; LV = left ventricle; AUC = area under curve; ACC = accuracy (%); CNN = convolutional neural network; HNN = hybrid neural network; MACE = major adverse cardiac events; MBF = myocardial blood flow; MPR = myocardial perfusion reserve; PSIR = phase-sensitive inversion recovery; ICC = c-index; R2 = correlation; SDAE = stack denoising autoencoder; SCDA = arrhythmic sudden cardiac death; HR = hazard ratio; CSAI = compressed sensing artificial intelligence; FCM-DNN = fuzzy C-means clustering combined with deep neural network; DL-CS = deep learning-constrained compressed sensing; GB-GAM = gradient boosting generalized additive model; NR = Not reported.

**Table 3:** Radiomics studies in CMR image in CAD.

Reference	# Subject (M/F)	Age(y) Mean ± Std	CMR Seq.	Target	AI Model	Performance
Arian, et al. [74]	43 (34/39)	58 ± 11	LGE	Myo function	SCAD- penalized SVM RP algorithm	AUC = 0.78 AUC = 0.65
Avard, et al. [75]	72 (NR)	NR	non- contrast bSSFP	MI Viable Normal	LR SVM	AUC = 0.93 ACC = 86.00 AUC = 0.92 ACC = 85.00
Ma, et al. [86]	68 (57/11)	55 ± 10	non- contrast T1-maps	MVO SLS	T1values+ RS	MVO: AUC = 0.86 SLS: AUC = 0.77
Abdulkareem, et al. [73]	272 (NR)	NR	bSSFP LGE	Segment Myo+MIS	SVM DT	AUC = 0.58 AUC = 0.57
Larroza, et al. [84]	50 (45/5)	61 ± 12	bSSFP LGE (2D+t)	Nonviable Viable Remote segments	RBF-SVM classifier	AUC = 0.84
Liu, et al. [85]	167 (149/18)	52 ± 11	LGE	MVO +/−	LASSO	AUC = 0.78
Frøysa, et al. [80]	52 (40/12)	64	LGE	MIS size	The texture- based probability mapping method	DSC = 0.69
Durmaz, et al. [79]	60 (55/5)	MACE: 57 ± 9 No MACE: 55 ± 9	LGE	MACE +/−	NN	AUC = 0.96 ACC = 89.40
Raisi- Estabragh, et al. [89]	92 (56/36)	NR	Perfusion	Rest and stress radiomics features	Model 4- Per territory (delta to histogram)	Sen = 53.00 Spec = 86.00
Khozeimeh, et al. [81]	63648 (NR)	NR	LGE Perfusion T2-w bSSFP	CAD +/−	Ensemble of CNNs and RF+Adam (optimizer)	AUC = 0.99 ACC = 99.18
Di Noto, et al. [87]	173 (153/20)	66 ± 9	LGE	MI Myo- carditis	SVM: (2D Features + RFE) LDA: (3D Features + PCA)	ACC = 88.00 ACC = 85.00
Kotu, et al. [82]	54 (NR)	NR	LGE	Arrhythmic risk	Several built-in classification schemes from matrix laboratory (matlab)	AUC = 0.96 ACC = 94.44
Rauseo, et al. [90]	2457 (NR)	HS 59 ± 7 CAD 67 ± 6	bSSFP	CAD CVD	SVM	IHD: AUC = 0.82 CVD: AUC = 0.79 MI: AUC = 0.87 IS: AUC = 0.81
Larroza, et al. [83]	44 (40/4)	61 ± 9	LGE bSSFP	Acute MI Chronic MI	SVM + poly	AUC = 0.86 AUC = 0.82
Baessler, et al. [76]	180 (138/42)	HS 48 ± 17 CAD 64 ± 10	Non- contrast bSSFP	Subacute MI Chronic MI	LR	Teta 1: AUC = 0.93 Perc.01: AUC = 0.92
Pujadas, et al. [88]	819 (NR)	66 ± 7	bSSFP	MI Other vascular pathologies	SVM	AUC = 0.76 ACC = 71.00
Wang, et al. [91]	115 (NR)	NR	LGE T1-w- transverse sBTFE T1+sBTFE	CAD +/−	Lasso RF/LR	AUC = 0.93 ACC = 0.93
Vande Berg, et al. [77]	148 (NR)	HS 48 ± 12 CAD 58 ± 12	Cine bSSFP T2w	CAD +/−	Lasso	ES: ACC = 0.84 ED: ACC = 0.76
Deng, et al. [78]	115 (89/26)	58 ± 11	Cine	CAD +/−	GNB	AUC = 0.91

# = number; CAD = coronary artery disease; HS = Healthy Subject; MI = myocardial infarction; MIS = myocardial infarction scar; HS = healthy subjects; Myo = myocardium; MVO = microvascular obstruction; MACE = major adverse cardiac events; bSSFP = balanced steady-state free precession; SVM = Support vector machine; poly = polynomial kernel; LR = logistic regression; DT = decision tree; LGE = late gadolinium enhancement; CMR = cardiac magnetic resonance; LV = left ventricle; AUC = area under curve; ACC = accuracy (%); DSC = dice similarity coefficient; Sen = sensitivity (%); Spec = specificity (%); RFE = recursive feature elimination; PCA = principal component analysis; CVD = cerebrovascular disease; IHD = ischemic heart dis-ease; SLS = segmental longitudinal strain; LDA = linear discriminant analysis; CNN = convolutional neural net-work; SCAD = smoothly clipped absolute deviation; RP = recursive partitioning; RS = radiomics signature; RBF = radial basis function; GNB = gaussian naive bayes; NR = Not reported.

## Data Availability

Data supporting the reported results are available from the corresponding author upon request.

## Supplementary Materials

The following supporting information can be downloaded at: https://www.mdpi.com/article/10.3390/jcdd12090345/s1, Table S1: CLAIM Quality.

## Abbreviations

The following abbreviations are used in this manuscript:

AI	Artificial intelligence
CAD	Coronary artery disease
MI	Myocardial infarction
CMR	Cardiac magnetic resonance
CTA	Computed tomography angiogram
LGE	Late gadolinium enhancement
LVEF	Left ventricular ejection fraction
ML	Machine learning
DL	Deep learning
SVM	Support vector machine
MACE	Major adverse cardiovascular events
ACC	Accuracy
AUC	Area under curve
LVR	Left ventricular remodelling
AHA	American Heart Association
SDAE	Stack denoising autoencode
CNN	Convolutional neural network
