# Supplementary material for: AI Applied to Cardiac Magnetic Resonance for Precision Medicine in Coronary Artery Disease: A Systematic Review
Source: J Cardiovasc Dev Dis. 2025 Sep 9;12(9):345. doi: 10.3390/jcdd12090345 (PMC12470487; doi:10.3390/jcdd12090345)
Supplement: Supplementary file 1 [file jcdd-12-00345-s001.zip › jcdd-3777001-supplementary.pdf]

| Section / Topic | | TITLE / ABSTRACT | ABSTRACT | INTRODUCTION | | METHODS | | | | | | | | | | | | | | | | | | | | | | | | | | | | | | RESULTS | | | | | DISCUSSION | | OTHER INFORMATION | | |
|---|---|---|---|---|---|---|---|---|---|---|---|---|---|---|---|---|---|---|---|---|---|---|---|---|---|---|---|---|---|---|---|---|---|---|---|---|---|---|---|---|---|---|---|---|---|
| | | | | | | *Study Design* | | *Data* | | | | | | | *Reference Standard* | | | | | *Data Partitions* | | *Testing Dat* | *Model* | | | *Training* | | | *Evaluation* | | | | | | | *Data* | | Model performance | | | | | | | |
| No. | | 1 | 2 | 3 | 4 | 5 | 6 | 7 | 8 | 9 | 10 | 11 | 12 | 13 | 14 | 15 | 16 | 17 | 18 | 19 | 20 | 21 | 22 | 23 | 24 | 25 | 26 | 27 | 28 | 29 | 30 | 31 | 32 | 33 | 34 | 35 | 36 | 37 | 38 | 39 | 40 | 41 | 42 | 43 | 44 |
| 1 | **Bekheet et. al, 2024** | Yes | Yes | Yes | Yes | Retro | Yes | Yes | Yes | Yes | Yes | Yes | Yes | Yes | Yes | Yes | No | Yes | Yes | Yes | Yes | Yes | Yes | Yes | Yes | Yes | Yes | Yes | Yes | Yes | Yes | No | Yes | No | No | Yes | No | Yes | Yes | Yes | Yes | Yes | Yes | Yes | Yes |
| 2 | **Lalande et al, 2022** | Yes | Yes | Yes | Yes | Retro | Yes | Yes | Yes | Yes | Yes | Yes | NA | NA | Yes | Yes | Yes | NA | No | Yes | Yes | Yes | Yes | NA | No | Yes | Yes | Yes | Yes | Yes | Yes | No | Yes | No | No | Yes | Yes | Yes | Yes | Yes | Yes | Yes | Yes | Yes | Yes |
| 3 | **Chen Z et. al, 2022** | Yes | Yes | Yes | Yes | Retro | Yes | Yes | Yes | Yes | Yes | Yes | NA | NA | Yes | Yes | Yes | NA | No | Yes | Yes | Yes | Yes | Yes | Yes | Yes | Yes | NA | Yes | Yes | Yes | No | Yes | No | No | Yes | Yes | Yes | Yes | Yes | Yes | Yes | Yes | Yes | Yes |
| 4 | **Muthulakshmi et. al, 2019** | Yes | Yes | Yes | Yes | N/A | Yes | Yes | Yes | Yes | Yes | Yes | NA | NA | Yes | Yes | Yes | NA | No | Yes | Yes | Yes | Yes | Yes | Yes | Yes | Yes | Yes | Yes | Yes | Yes | No | Yes | No | No | Yes | No | Yes | Yes | Yes | No | Yes | Yes | Yes | Yes |
| 5 | **Xu et. al, 2019** | Yes | Yes | Yes | Yes | N/A | Yes | Yes | Yes | Yes | Yes | NA | Yes | Yes | Yes | Yes | No | NA | No | No | No | Yes | Yes | Yes | No | Yes | Yes | Yes | Yes | Yes | Yes | No | Yes | No | No | Yes | Yes | Yes | Yes | Yes | Yes | Yes | No | Yes | Yes |
| 6 | **Attallah et al, 2023** | Yes | Yes | Yes | Yes | Retro | Yes | Yes | Yes | Yes | Yes | Yes | NA | NA | Yes | Yes | Yes | NA | No | Yes | Yes | Yes | Yes | Yes | Yes | Yes | Yes | Yes | Yes | Yes | Yes | No | Yes | No | No | Yes | No | Yes | Yes | Yes | Yes | Yes | Yes | Yes | Yes |
| 7 | **Zhang et. al, 2019** | Yes | Yes | Yes | Yes | Retro | Yes | Yes | Yes | Yes | Yes | NA | NA | Yes | Yes | Yes | Yes | NA | No | Yes | Yes | Yes | Yes | Yes | Yes | Yes | Yes | Yes | Yes | Yes | Yes | No | Yes | No | No | Yes | Yes | Yes | Yes | Yes | Yes | Yes | Yes | Yes | Yes |
| 8 | **Joloudari et. al, 2022** | Yes | Yes | Yes | Yes | Retro | Yes | Yes | Yes | Yes | Yes | Yes | No | Yes | Yes | Yes | Yes | NA | No | Yes | Yes | Yes | Yes | Yes | Yes | Yes | Yes | Yes | Yes | Yes | Yes | No | Yes | No | No | Yes | No | Yes | Yes | Yes | Yes | Yes | Yes | Yes | Yes |
| 9 | **Iqbal et. al, 2024** | Yes | Yes | Yes | Yes | N/A | Yes | Yes | Yes | Yes | Yes | NA | NA | Yes | Yes | Yes | Yes | NA | No | Yes | Yes | Yes | Yes | Yes | No | Yes | Yes | Yes | Yes | Yes | Yes | Yes | Yes | No | No | Yes | No | Yes | Yes | Yes | Yes | Yes | Yes | Yes | Yes |
| 10 | **Wu2 et al., 2023** | Yes | Yes | Yes | Yes | Prosp | Yes | Yes | Yes | Yes | Yes | No | NA | Yes | Yes | Yes | Yes | Yes | No | Yes | No | Yes | No | No | No | No | Yes | No | Yes | Yes | Yes | No | Yes | No | No | Yes | Yes | Yes | Yes | Yes | Yes | Yes | Yes | Yes | Yes |
| 11 | **Chen, 2021** | Yes | Yes | Yes | Yes | N/A | Yes | Yes | Yes | Yes | Yes | No | No | Yes | Yes | Yes | Yes | Yes | No | No | No | Yes | Yes | Yes | NA | No | Yes | No | Yes | Yes | Yes | No | Yes | No | No | Yes | No | Yes | Yes | Yes | No | Yes | Yes | Yes | Yes |
| 12 | **Paciorek et. al 2024** | Yes | Yes | Yes | Yes | Retro | Yes | Yes | Yes | Yes | Yes | No | No | Yes | Yes | Yes | Yes | No | No | Yes | Yes | Yes | Yes | Yes | Yes | Yes | Yes | Yes | Yes | Yes | Yes | Yes | Yes | Yes | No | Yes | Yes | Yes | Yes | Yes | Yes | Yes | Yes | Yes | Yes |
| 13 | **Backhaus et. al, 2022** | Yes | Yes | Yes | Yes | Prosp | Yes | Yes | Yes | Yes | Yes | No | No | Yes | Yes | Yes | Yes | Yes | No | Yes | Yes | Yes | Yes | Yes | No | No | Yes | Yes | Yes | Yes | Yes | No | Yes | No | Yes | Yes | Yes | Yes | Yes | Yes | Yes | Yes | Yes | Yes | Yes |
| 14 | **Schuster et. al, 2020** | Yes | Yes | Yes | Yes | Retro | Yes | Yes | Yes | Yes | Yes | No | No | Yes | Yes | Yes | Yes | No | No | No | No | Yes | Yes | Yes | Yes | No | Yes | Yes | Yes | Yes | Yes | No | Yes | No | No | Yes | Yes | Yes | Yes | Yes | Yes | Yes | Yes | Yes | Yes |
| 15 | **Pezel et al., 2023** | Yes | Yes | Yes | Yes | Retro | Yes | Yes | Yes | Yes | Yes | NA | No | Yes | Yes | Yes | Yes | Yes | No | Yes | No | Yes | Yes | Yes | No | No | Yes | No | Yes | Yes | Yes | No | Yes | Yes | No | Yes | Yes | Yes | Yes | Yes | Yes | Yes | Yes | Yes | Yes |
| 16 | **Knott et. al, 2020** | Yes | Yes | Yes | Yes | N/A | Yes | Yes | Yes | Yes | Yes | No | No | Yes | Yes | Yes | No | Yes | No | No | No | Yes | No | No | No | No | NA | No | Yes | Yes | Yes | No | Yes | No | No | Yes | Yes | Yes | Yes | Yes | Yes | Yes | Yes | Yes | Yes |
| 17 | **Popescu et. al, 2022** | Yes | Yes | Yes | Yes | Prosp | Yes | Yes | Yes | Yes | Yes | NA | NA | Yes | Yes | Yes | Yes | Yes | No | Yes | Yes | Yes | Yes | Yes | Yes | Yes | Yes | Yes | Yes | Yes | Yes | Yes | Yes | Yes | No | Yes | Yes | Yes | Yes | Yes | Yes | Yes | Yes | Yes | Yes |
| 18 | **Pezel et. al, 2022** | Yes | Yes | Yes | Yes | Retro | Yes | Yes | Yes | Yes | Yes | NA | NA | Yes | Yes | Yes | Yes | Yes | No | Yes | Yes | Yes | Yes | Yes | Yes | Yes | Yes | Yes | Yes | Yes | Yes | Yes | Yes | Yes | No | Yes | Yes | Yes | Yes | Yes | Yes | Yes | Yes | Yes | Yes |
| 19 | **Maleckar et. al, 2021** | Yes | Yes | Yes | Yes | N/A | Yes | Yes | Yes | Yes | Yes | No | No | No | Yes | Yes | Yes | NA | No | Yes | Yes | Yes | Yes | Yes | Yes | Yes | Yes | Yes | Yes | Yes | Yes | Yes | Yes | No | No | Yes | No | Yes | Yes | Yes | Yes | Yes | Yes | Yes | Yes |
| 20 | **Ghanbari et. al, 2023** | Yes | Yes | Yes | Yes | Retro | Yes | Yes | Yes | No | Yes | No | No | Yes | Yes | Yes | Yes | NA | Yes | Yes | Yes | Yes | Yes | Yes | Yes | Yes | Yes | Yes | Yes | Yes | Yes | Yes | Yes | Yes | Yes | Yes | Yes | Yes | Yes | Yes | Yes | Yes | No | No | Yes |
| 21 | **Okada et al., 2020** | Yes | Yes | Yes | Yes | Retro | Yes | Yes | Yes | Yes | Yes | No | No | Yes | Yes | Yes | Yes | NA | No | Yes | No | Yes | Yes | Yes | No | Yes | Yes | Yes | Yes | Yes | Yes | Yes | Yes | No | No | Yes | Yes | Yes | Yes | Yes | Yes | Yes | Yes | Yes | Yes |
| 22 | **Zaidi et. al, 2023** | Yes | Yes | Yes | Yes | Prosp | Yes | Yes | Yes | Yes | Yes | No | No | Yes | Yes | Yes | Yes | Yes | No | No | Yes | Yes | Yes | NA | Yes | NA | NA | NA | Yes | Yes | Yes | Yes | Yes | NA | NA | Yes | Yes | Yes | Yes | Yes | Yes | Yes | Yes | NA | Yes |
| 23 | **Chen B. et. al, 2023** | Yes | Yes | Yes | Yes | Prosp | Yes | Yes | Yes | Yes | Yes | No | No | Yes | Yes | Yes | Yes | Yes | Yes | Yes | Yes | Yes | Yes | Yes | Yes | Yes | Yes | Yes | Yes | Yes | Yes | Yes | Yes | Yes | Yes | Yes | Yes | Yes | Yes | Yes | Yes | Yes | No | No | Yes |
| 24 | **Chen M. et. al, 2019** | Yes | Yes | Yes | Yes | N/A | Yes | Yes | Yes | Yes | Yes | No | No | Yes | Yes | Yes | Yes | Yes | No | Yes | Yes | Yes | Yes | Yes | Yes | Yes | Yes | Yes | Yes | Yes | Yes | No | Yes | No | No | Yes | Yes | Yes | Yes | Yes | No | Yes | No | No | Yes |
| 25 | **Feng Y et. al, 2018** | Yes | Yes | Yes | Yes | N/A | Yes | Yes | Yes | Yes | Yes | No | No | Yes | Yes | Yes | Yes | Yes | No | Yes | Yes | Yes | Yes | Yes | Yes | Yes | Yes | Yes | Yes | Yes | Yes | No | Yes | No | No | Yes | No | Yes | Yes | Yes | Yes | Yes | No | No | Yes |
| 26 | **Kim et. al, 2024** | Yes | Yes | Yes | Yes | Retro | Yes | Yes | Yes | No | Yes | No | No | Yes | Yes | Yes | Yes | Yes | Yes | Yes | Yes | Yes | Yes | Yes | Yes | Yes | Yes | Yes | Yes | Yes | Yes | Yes | Yes | Yes | Yes | Yes | No | Yes | Yes | Yes | Yes | Yes | No | No | Yes |
| 27 | **Wang et. al, 2023** | Yes | Yes | Yes | Yes | N/A | Yes | Yes | Yes | Yes | Yes | No | No | Yes | Yes | Yes | Yes | Yes | No | Yes | Yes | Yes | Yes | Yes | Yes | Yes | Yes | Yes | Yes | Yes | Yes | No | Yes | No | No | Yes | No | Yes | Yes | Yes | Yes | Yes | No | Yes | Yes |
| 28 | **Hernández-Casillas et. al, 2022** | Yes | Yes | Yes | Yes | N/A | Yes | Yes | Yes | Yes | Yes | No | No | Yes | No | No | No | NA | No | Yes | No | Yes | Yes | Yes | Yes | Yes | Yes | Yes | Yes | Yes | Yes | No | Yes | No | No | Yes | No | Yes | Yes | Yes | No | Yes | No | No | Yes |
| 29 | **Mauger et. al, 2023** | Yes | Yes | Yes | Yes | Prosp | Yes | Yes | Yes | Yes | Yes | NA | NA | Yes | Yes | Yes | No | No | No | Yes | Yes | Yes | Yes | Yes | Yes | Yes | Yes | Yes | Yes | Yes | Yes | No | Yes | No | No | Yes | Yes | Yes | Yes | Yes | Yes | Yes | No | No | Yes |
| 30 | **Dieu et. al, 2022** | Yes | Yes | Yes | Yes | Retro | Yes | Yes | Yes | Yes | Yes | NA | NA | No | Yes | Yes | Yes | Yes | No | Yes | Yes | Yes | Yes | Yes | Yes | Yes | Yes | Yes | Yes | Yes | Yes | Yes | Yes | No | No | Yes | No | Yes | Yes | Yes | Yes | Yes | Yes | Yes | Yes |
| 31 | **Böttcher et. al, 2020** | Yes | Yes | Yes | Yes | Retro | Yes | Yes | Yes | Yes | Yes | NA | No | Yes | Yes | Yes | Yes | Yes | No | Yes | No | Yes | Yes | No | No | No | Yes | Yes | Yes | Yes | Yes | Yes | Yes | No | No | Yes | Yes | Yes | Yes | Yes | Yes | Yes | Yes | Yes | Yes |
| 32 | **Goldfarb et. al 2019** | Yes | Yes | Yes | Yes | Retro | Yes | Yes | Yes | Yes | Yes | No | No | Yes | Yes | Yes | Yes | Yes | Yes | Yes | Yes | Yes | Yes | No | Yes | Yes | Yes | Yes | Yes | Yes | Yes | No | Yes | No | No | Yes | No | Yes | Yes | Yes | Yes | Yes | Yes | Yes | Yes |
| 33 | **Wu et al., 2023** | Yes | Yes | Yes | Yes | Prosp | Yes | Yes | Yes | Yes | Yes | No | No | Yes | Yes | Yes | Yes | Yes | Yes | Yes | No | Yes | Yes | Yes | Yes | Yes | Yes | Yes | Yes | Yes | Yes | No | Yes | No | No | Yes | Yes | Yes | Yes | Yes | Yes | Yes | Yes | Yes | Yes |
| 34 | **Cau et al, 2024** | Yes | Yes | Yes | Yes | Retro | Yes | Yes | Yes | Yes | Yes | No | Yes | Yes | Yes | Yes | Yes | Yes | Yes | Yes | Yes | Yes | Yes | Yes | Yes | Yes | Yes | Yes | Yes | Yes | Yes | No | Yes | No | No | Yes | Yes | Yes | Yes | Yes | Yes | Yes | Yes | Yes | Yes |
| 35 | **Alskaf et. al, 2024** | Yes | Yes | Yes | Yes | Retro | Yes | Yes | Yes | Yes | Yes | No | No | Yes | Yes | Yes | Yes | Yes | Yes | Yes | Yes | Yes | Yes | Yes | Yes | Yes | Yes | Yes | Yes | Yes | Yes | No | Yes | No | No | Yes | Yes | Yes | Yes | Yes | Yes | Yes | Yes | Yes | Yes |
| 36 | **Alskaf et. al, 2025** | Yes | Yes | Yes | Yes | Retro | Yes | Yes | Yes | Yes | Yes | Yes | No | Yes | Yes | Yes | Yes | Yes | Yes | Yes | Yes | Yes | Yes | Yes | No | Yes | Yes | Yes | Yes | Yes | Yes | No | Yes | No | No | Yes | Yes | Yes | Yes | Yes | Yes | Yes | Yes | Yes | Yes |
| 37 | **Corral Acero et. al, 2024** | Yes | Yes | Yes | Yes | N/A | Yes | Yes | Yes | Yes | Yes | No | No | Yes | Yes | Yes | Yes | NA | No | No | No | No | Yes | Yes | No | Yes | Yes | No | Yes | Yes | Yes | Yes | Yes | No | Yes | Yes | No | Yes | Yes | No | Yes | Yes | Yes | Yes | Yes |
| 38 | **Li et. al, 2024** | Yes | Yes | Yes | Yes | Retro | Yes | Yes | Yes | Yes | Yes | No | No | Yes | Yes | Yes | Yes | Yes | Yes | No | No | Yes | Yes | Yes | No | No | Yes | Yes | Yes | Yes | Yes | No | Yes | No | No | Yes | Yes | Yes | Yes | No | Yes | Yes | Yes | Yes | Yes |
| 39 | **Udin et. al, 2024** | Yes | Yes | Yes | Yes | Retro | Yes | Yes | Yes | Yes | Yes | No | No | Yes | Yes | Yes | Yes | Yes | Yes | Yes | Yes | Yes | Yes | Yes | Yes | Yes | Yes | Yes | Yes | Yes | Yes | No | Yes | Yes | No | Yes | Yes | Yes | Yes | Yes | Yes | Yes | Yes | Yes | Yes |
| 40 | **Frøysa et. al, 2025** | Yes | Yes | Yes | Yes | Retro | Yes | Yes | Yes | No | No | No | No | Yes | Yes | Yes | Yes | No | Yes | No | No | Yes | Yes | No | Yes | No | No | Yes | No | Yes | No | No | Yes | No | No | Yes | Yes | Yes | Yes | Yes | Yes | Yes | Yes | Yes | Yes |
| 41 | **GhaffariJolfayi et. al, 2025** | Yes | Yes | Yes | Yes | Retro | Yes | Yes | Yes | Yes | Yes | No | No | Yes | Yes | Yes | Yes | Yes | Yes | Yes | Yes | Yes | Yes | Yes | Yes | Yes | Yes | NA | Yes | Yes | Yes | No | Yes | No | No | Yes | Yes | Yes | Yes | Yes | Yes | Yes | Yes | Yes | Yes |
| 42 | **Jacob et. al, 2025** | Yes | Yes | Yes | Yes | Retro | Yes | Yes | Yes | Yes | Yes | No | Yes | Yes | Yes | Yes | Yes | Yes | Yes | Yes | Yes | Yes | Yes | Yes | Yes | Yes | Yes | Yes | Yes | Yes | Yes | No | Yes | No | No | Yes | Yes | Yes | Yes | Yes | Yes | Yes | Yes | No | Yes |
| 43 | **Righetti et. al, 2025** | Yes | Yes | Yes | Yes | Retro | Yes | Yes | Yes | Yes | Yes | No | Yes | Yes | Yes | Yes | Yes | Yes | Yes | Yes | Yes | Yes | Yes | Yes | Yes | Yes | Yes | Yes | Yes | Yes | Yes | No | Yes | No | No | Yes | Yes | Yes | Yes | Yes | Yes | Yes | Yes | Yes | Yes |
| 44 | **Wu et. al, 2024** | Yes | Yes | Yes | Yes | Prosp | Yes | Yes | Yes | Yes | Yes | No | Yes | Yes | Yes | Yes | Yes | Yes | Yes | No | No | No | Yes | Yes | No | No | Yes | No | Yes | Yes | No | No | Yes | No | No | Yes | Yes | Yes | Yes | No | Yes | Yes | Yes | Yes | Yes |
| 45 | **Guglielmo et. al, 2024** | Yes | Yes | Yes | Yes | Retro | Yes | Yes | Yes | Yes | Yes | No | Yes | Yes | Yes | Yes | Yes | Yes | Yes | Yes | Yes | Yes | Yes | Yes | Yes | Yes | Yes | Yes | Yes | Yes | Yes | No | Yes | No | No | Yes | Yes | Yes | Yes | Yes | Yes | Yes | Yes | Yes | Yes |
| 46 | **Pezel et. al, 2025** | Yes | Yes | Yes | Yes | Retro | Yes | Yes | Yes | Yes | Yes | No | Yes | Yes | Yes | Yes | Yes | Yes | Yes | Yes | Yes | Yes | Yes | Yes | Yes | Yes | Yes | Yes | Yes | Yes | No | No | Yes | Yes | No | Yes | Yes | Yes | Yes | Yes | Yes | Yes | Yes | Yes | Yes |
| | | | | | | | | | | | | | | | | | | | | | | | | | | | | | | | | | | | | | | | | | | | | | |
| 1 | **Gröschel, 2024** | No | Yes | Yes | Yes | Retro | Yes | Yes | Yes | Yes | Yes | No | NA | Yes | Yes | Yes | Yes | Yes | No | No | No | Yes | Yes | Yes | No | Yes | No | Yes | Yes | Yes | Yes | Yes | Yes | Yes | No | Yes | Yes | Yes | Yes | Yes | Yes | Yes | No | Yes | Yes |
| 2 | **Mosquera-Rojas et. al, 2023** | Yes | Yes | Yes | Yes | Retro | Yes | Yes | Yes | Yes | Yes | No | No | Yes | Yes | Yes | Yes | Yes | Yes | Yes | Yes | Yes | Yes | Yes | Yes | Yes | Yes | Yes | Yes | Yes | Yes | Yes | Yes | Yes | Yes | Yes | Yes | Yes | Yes | Yes | Yes | Yes | No | No | Yes |
| 3 | **Barbaroux et. al, 2023** | Yes | Yes | Yes | Yes | Retro | Yes | Yes | Yes | Yes | Yes | No | No | Yes | Yes | Yes | Yes | Yes | Yes | Yes | Yes | Yes | Yes | Yes | Yes | Yes | Yes | Yes | Yes | Yes | Yes | Yes | Yes | Yes | Yes | Yes | Yes | Yes | Yes | Yes | Yes | Yes | Yes | Yes | Yes |
| 4 | **Yan et. al, 2022** | Yes | Yes | Yes | Yes | N/A | Yes | Yes | Yes | Yes | Yes | No | No | NA | NA | NA | NA | NA | NA | Yes | Yes | Yes | Yes | Yes | Yes | Yes | Yes | Yes | Yes | Yes | Yes | No | Yes | No | No | Yes | No | Yes | Yes | Yes | Yes | Yes | No | No | Yes |
| 5 | **Scannell, et. al, 2020** | Yes | Yes | Yes | Yes | Retro | Yes | Yes | Yes | Yes | Yes | No | No | Yes | NA | NA | NA | NA | NA | Yes | Yes | Yes | Yes | Yes | Yes | Yes | Yes | Yes | Yes | Yes | Yes | No | Yes | No | No | Yes | No | Yes | Yes | Yes | Yes | Yes | Yes | No | Yes |
| 6 | **Ahmad et. al, 2022** | Yes | Yes | Yes | Yes | N/A | Yes | Yes | Yes | Yes | No | No | No | Yes | No | Yes | No | No | No | Yes | No | No | Yes | Yes | No | No | NA | Yes | Yes | Yes | Yes | Yes | No | No | No | Yes | Yes | Yes | Yes | Yes | No | Yes | No | No | Yes |
| 7 | **Kim et. al, 2020** | Yes | Yes | Yes | Yes | N/A | Yes | Yes | Yes | Yes | Yes | No | No | Yes | No | NA | NA | NA | NA | Yes | Yes | Yes | Yes | Yes | Yes | Yes | Yes | Yes | Yes | Yes | Yes | No | Yes | No | No | Yes | No | Yes | Yes | Yes | Yes | Yes | No | No | Yes |
| 8 | **Liu et. al, 2019** | Yes | Yes | Yes | Yes | N/A | Yes | Yes | Yes | Yes | No | No | No | Yes | Yes | Yes | Yes | No | No | Yes | Yes | Yes | Yes | Yes | Yes | Yes | Yes | Yes | Yes | Yes | Yes | No | Yes | No | No | Yes | No | Yes | Yes | Yes | Yes | Yes | Yes | No | Yes |

| | | | | | | | | | | | | | | | | | | | | | | | | | | | | | | | | | | | | | | | | | | | | | | | | | | | |
|---|---|---|---|---|---|---|---|---|---|---|---|---|---|---|---|---|---|---|---|---|---|---|---|---|---|---|---|---|---|---|---|---|---|---|---|---|---|---|---|---|---|---|---|---|---|---|---|---|---|---|---|
| 9 | Tan et. al, 2018 | Yes |  | Yes |  | Yes | Yes |  | N/A | Yes | Yes | Yes | Yes | Yes | No | No | Yes | Yes | Yes | Yes | No | No | Yes | Yes | Yes | Yes | Yes | Yes | Yes | Yes | Yes | Yes | Yes | Yes | No | Yes | No | No |  | Yes | No | Yes | Yes | Yes |  | Yes | Yes |  | No | No | Yes |
| 10 | Chen Y. et. al, 2022 (Myocardial...) | Yes |  | Yes |  | Yes | Yes |  | N/A | Yes | Yes | Yes | Yes | Yes | No | No | Yes | Yes | No | No | No | No | Yes | Yes | Yes | Yes | Yes | Yes | Yes | Yes | Yes | Yes | Yes | Yes | No | Yes | No | No |  | Yes | No | Yes | Yes | Yes |  | No | Yes |  | Yes | Yes | Yes |
| 11 | Papetti et. al, 2023 | Yes |  | Yes |  | Yes | Yes |  | Retro | Yes | Yes | Yes | Yes | Yes | No | No | Yes | Yes | Yes | Yes | No | No | Yes | Yes | Yes | Yes | Yes | Yes | Yes | Yes | Yes | Yes | Yes | Yes | No | Yes | No | No |  | Yes | No | Yes | Yes | Yes |  | Yes | Yes |  | Yes | No | Yes |
| 12 | Lecesne et. al, 2023 | Yes |  | Yes |  | Yes | Yes |  | N/A | Yes | Yes | Yes | Yes | Yes | No | No | No | Yes | Yes | Yes | Yes | Yes | Yes | Yes | Yes | Yes | Yes | Yes | Yes | Yes | Yes | Yes | Yes | Yes | No | Yes | No | No |  | Yes | No | Yes | Yes | Yes |  | Yes | Yes |  | No | NA | Yes |
| 13 | Lin et. al, 2022 | Yes |  | Yes |  | Yes | Yes |  | N/A | Yes | Yes | Yes | Yes | Yes | No | No | Yes | Yes | Yes | Yes | No | No | Yes | Yes | Yes | Yes | Yes | Yes | Yes | Yes | Yes | Yes | Yes | Yes | No | Yes | No | No |  | Yes | No | Yes | Yes | Yes |  | Yes | Yes |  | No | No | Yes |
| 14 | Mamalakis et. al, 2023 | Yes |  | Yes |  | Yes | Yes |  | N/A | Yes | Yes | Yes | Yes | Yes | No | No | Yes | Yes | Yes | NA | Yes | No | Yes | Yes | Yes | Yes | NA | No | Yes | Yes | NA | Yes | Yes | Yes | Yes | Yes | Yes | NA |  | Yes | No | Yes | Yes | No |  | Yes | Yes |  | Yes | NA | Yes |
| 15 | Xu Ch. et. al, 2023 | Yes |  | Yes |  | Yes | Yes |  | Retro | Yes | Yes | Yes | Yes | NA | Yes | NA | Yes | Yes | Yes | Yes | Yes | Yes | Yes | Yes | Yes | Yes | NA | Yes | Yes | Yes | NA | Yes | Yes | Yes | Yes | Yes | Yes | NA |  | Yes | No | Yes | Yes | NA |  | No | Yes |  | Yes | NA | Yes |
| 16 | Chen et. al, 2022 (Automatic...) | Yes |  | Yes |  | Yes | Yes |  | N/A | Yes | Yes | Yes | Yes | Yes | No | No | Yes | Yes | Yes | Yes | Yes | Yes | Yes | Yes | Yes | Yes | Yes | Yes | Yes | Yes | Yes | Yes | Yes | Yes | Yes | Yes | No | No |  | Yes | No | Yes | Yes | Yes |  | Yes | Yes |  | No | NA | Yes |
| 17 | Heidenreich et. al, 2021 | Yes |  | Yes |  | Yes | Yes |  | Retro | Yes | Yes | Yes | Yes | Yes | No | No | Yes | Yes | Yes | Yes | Yes | No | Yes | Yes | Yes | Yes | Yes | Yes | Yes | Yes | Yes | Yes | Yes | Yes | Yes | Yes | No | No |  | Yes | Yes | Yes | Yes | Yes |  | Yes | Yes |  | No | No | Yes |
| 18 | Xu et. al, 2020 | Yes |  | Yes |  | Yes | Yes |  | Retro | Yes | Yes | Yes | Yes | Yes | No | No | Yes | Yes | Yes | Yes | Yes | No | Yes | No | Yes | Yes | Yes | Yes | Yes | Yes | Yes | Yes | Yes | Yes | Yes | Yes | No | No |  | Yes | No | Yes | Yes | Yes |  | Yes | Yes |  | No | No | Yes |
| 19 | Zabihollahy et. al, 2019 | Yes |  | Yes |  | Yes | Yes |  | N/A | Yes | Yes | Yes | Yes | Yes | No | No | Yes | Yes | No | No | No | No | Yes | Yes | Yes | Yes | Yes | Yes | Yes | Yes | Yes | Yes | Yes | Yes | Yes | Yes | Yes | No |  | Yes | Yes | Yes | Yes | Yes |  | Yes | Yes |  | No | No | Yes |
| 20 | Moccia et.al, 2019 | Yes |  | Yes |  | Yes | Yes |  | Retro | Yes | Yes | Yes | Yes | Yes | No | No | Yes | Yes | No | No | No | No | Yes | Yes | Yes | Yes | Yes | Yes | Yes | Yes | Yes | Yes | Yes | Yes | Yes | Yes | No | No |  | Yes | No | Yes | Yes | Yes |  | Yes | Yes |  | Yes | No | Yes |
| 21 | Li et. al, 2023 | Yes |  | Yes |  | Yes | Yes |  | N/A | Yes | Yes | Yes | Yes | Yes | No | No | Yes | Yes | Yes | Yes | Yes | Yes | Yes | Yes | Yes | Yes | Yes | Yes | Yes | Yes | Yes | Yes | Yes | Yes | Yes | Yes | Yes | Yes |  | Yes | No | Yes | Yes | No |  | No | Yes |  | Yes | Yes | Yes |
| 22 | Qiu et. al, 2023 | Yes |  | Yes |  | Yes | Yes |  | N/A | Yes | Yes | Yes | Yes | Yes | No | No | Yes | Yes | Yes | Yes | Yes | Yes | Yes | Yes | Yes | Yes | Yes | Yes | Yes | Yes | Yes | Yes | Yes | Yes | Yes | Yes | Yes | Yes |  | Yes | No | Yes | Yes | No |  | Yes | Yes |  | Yes | Yes | Yes |
| 23 | Cui H. et al, 2022 | Yes |  | Yes |  | Yes | Yes |  | N/A | Yes | Yes | Yes | Yes | Yes | NA | NA | NA | Yes | Yes | Yes | NA | NA | No | Yes | Yes | Yes | NA | No | Yes | Yes | Yes | Yes | No | No | Yes | Yes | No | NA |  | Yes | No | Yes | Yes | No |  | No | Yes |  | Yes | NA | Yes |
| 24 | Li et. al, 2022 | Yes |  | Yes |  | Yes | Yes |  | N/A | Yes | Yes | Yes | Yes | Yes | NA | NA | NA | Yes | Yes | Yes | Yes | NA | Yes | No | Yes | Yes | Yes | Yes | Yes | Yes | Yes | Yes | Yes | Yes | No | Yes | No | No |  | Yes | No | Yes | Yes | Yes |  | No | Yes |  | Yes | Yes | Yes |
| 25 | Cui et. al, 2022 | Yes |  | Yes |  | Yes | Yes |  | N/A | Yes | Yes | Yes | Yes | Yes | NA | NA | NA | Yes | Yes | Yes | Yes | NA | Yes | Yes | Yes | Yes | Yes | Yes | Yes | Yes | Yes | Yes | Yes | Yes | No | Yes | No | No |  | Yes | No | Yes | Yes | Yes |  | No | Yes |  | No | No | Yes |
| 26 | Brahim et. al, 2022 | Yes |  | Yes |  | Yes | Yes |  | Retro | Yes | Yes | Yes | Yes | Yes | NA | NA | NA | Yes | Yes | Yes | Yes | NA | Yes | Yes | Yes | Yes | Yes | Yes | Yes | Yes | Yes | Yes | Yes | Yes | No | Yes | No | No |  | Yes | No | Yes | Yes | Yes |  | No | Yes |  | Yes | Yes | Yes |
| 27 | de la Rosa et. al, 2021 | Yes |  | Yes |  | Yes | Yes |  | N/A | Yes | Yes | Yes | Yes | Yes | No | No | Yes | Yes | Yes | Yes | Yes | Yes | Yes | Yes | Yes | Yes | Yes | Yes | Yes | Yes | Yes | Yes | Yes | Yes | No | Yes | No | No |  | Yes | No | Yes | Yes | Yes |  | Yes | Yes |  | No | Yes | Yes |
| 28 | Brahim et. al, 2021 | Yes |  | Yes |  | Yes | Yes |  | N/A | Yes | Yes | Yes | Yes | Yes | NA | NA | NA | Yes | Yes | Yes | Yes | NA | Yes | Yes | Yes | Yes | Yes | Yes | Yes | Yes | Yes | Yes | Yes | Yes | No | Yes | No | No |  | Yes | No | Yes | Yes | Yes |  | No | Yes |  | Yes | Yes | Yes |
| 29 | Chen S. et. al, 2023 | Yes |  | Yes |  | Yes | Yes |  | Retro | Yes | Yes | Yes | Yes | Yes | No | No | Yes | Yes | Yes | Yes | Yes | Yes | Yes | Yes | Yes | Yes | Yes | Yes | Yes | Yes | Yes | Yes | Yes | Yes | Yes | Yes | Yes | Yes |  | Yes | Yes | No | Yes | NA |  | Yes | Yes |  | NA | NA | Yes |
| 30 | Arega et. al, 2023 | Yes |  | Yes |  | Yes | Yes |  | N/A | Yes | Yes | Yes | Yes | Yes | NA | NA | Yes | Yes | Yes | Yes | Yes | Yes | Yes | Yes | Yes | Yes | Yes | Yes | Yes | Yes | Yes | Yes | Yes | Yes | Yes | Yes | Yes | Yes |  | Yes | No | Yes | Yes | No |  | Yes | Yes |  | Yes | Yes | Yes |
| 31 | Popescu et al., 2022 | Yes |  | Yes |  | Yes | Yes |  | N/A | Yes | Yes | Yes | Yes | Yes | NA | NA | Yes | Yes | Yes | Yes | Yes | No | Yes | Yes | Yes | Yes | Yes | Yes | Yes | Yes | Yes | Yes | Yes | Yes | No | Yes | No | No |  | Yes | No | Yes | Yes | Yes |  | Yes | Yes |  | Yes | Yes | Yes |
| 32 | Mamalakis et al., 2021 | Yes |  | Yes |  | Yes | Yes |  | N/A | Yes | Yes | Yes | Yes | Yes | NA | NA | NA | Yes | Yes | Yes | Yes | Yes | Yes | Yes | Yes | Yes | Yes | Yes | Yes | Yes | Yes | Yes | Yes | Yes | No | Yes | Yes | No |  | Yes | No | Yes | Yes | Yes |  | Yes | Yes |  | Yes | Yes | Yes |
| 33 | Al-antari, 2024 | Yes |  | Yes |  | Yes | Yes |  | Retro | Yes | Yes | Yes | Yes | Yes | NA | NA | NA | Yes | Yes | Yes | Yes | No | Yes | Yes | Yes | Yes | Yes | Yes | Yes | Yes | Yes | Yes | Yes | Yes | No | Yes | Yes | No |  | Yes | No | Yes | Yes | Yes |  | Yes | Yes |  | Yes | Yes | Yes |
| 34 | Jani et. al, 2024 | Yes |  | Yes |  | Yes | Yes |  | Prosp | Yes | Yes | Yes | Yes | Yes | No | Yes | Yes | Yes | Yes | Yes | Yes | Yes | Yes | Yes | Yes | Yes | Yes | Yes | Yes | Yes | Yes | Yes | Yes | Yes | No | Yes | Yes | No |  | Yes | Yes | Yes | Yes | Yes |  | Yes | Yes |  | Yes | Yes | Yes |
| 35 | Qi et. al, 2024 | Yes |  | Yes |  | Yes | Yes |  | Retro | Yes | Yes | Yes | Yes | Yes | No | Yes | Yes | Yes | Yes | Yes | Yes | Yes | Yes | Yes | Yes | Yes | Yes | Yes | Yes | Yes | Yes | Yes | Yes | Yes | No | Yes | Yes | No |  | Yes | Yes | Yes | Yes | Yes |  | Yes | Yes |  | Yes | Yes | Yes |
| 36 | Yalcinkaya et. al, 2024 | Yes |  | Yes |  | Yes | Yes |  | Retro | Yes | Yes | Yes | Yes | Yes | No | Yes | Yes | Yes | Yes | Yes | Yes | Yes | Yes | Yes | Yes | Yes | Yes | Yes | Yes | Yes | Yes | Yes | Yes | Yes | No | Yes | Yes | No |  | Yes | No | Yes | Yes | Yes |  | Yes | Yes |  | Yes | Yes | Yes |
| 37 | Lin et. al, 2025 | Yes |  | Yes |  | Yes | Yes |  | Retro | Yes | Yes | Yes | Yes | Yes | No | Yes | Yes | Yes | Yes | Yes | Yes | Yes | Yes | Yes | Yes | Yes | Yes | Yes | Yes | Yes | Yes | Yes | Yes | Yes | No | Yes | No | No |  | Yes | No | Yes | Yes | Yes |  | Yes | Yes |  | Yes | Yes | Yes |
| 38 | Ben Khalifa et. al, 2025 | Yes |  | Yes |  | Yes | Yes |  | Retro | Yes | Yes | Yes | Yes | Yes | No | Yes | Yes | Yes | Yes | Yes | Yes | Yes | Yes | Yes | Yes | Yes | Yes | Yes | Yes | Yes | Yes | Yes | Yes | Yes | No | Yes | No | No |  | Yes | No | Yes | Yes | Yes |  | Yes | Yes |  | Yes | Yes | Yes |
| 39 | Li et. al, 2025 | Yes |  | Yes |  | Yes | Yes |  | N/A | Yes | Yes | NA | Yes | Yes | NA | NA | No | Yes | Yes | Yes | Yes | Yes | Yes | Yes | Yes | Yes | Yes | Yes | Yes | Yes | Yes | Yes | Yes | Yes | No | NA | NA | NA |  | NA | NA | Yes | Yes | Yes |  | Yes | Yes |  | Yes | Yes | Yes |
| 40 | Bernardo et. al, 2024 | Yes |  | Yes |  | Yes | Yes |  | N/A | Yes | Yes | NA | Yes | Yes | NA | NA | No | Yes | Yes | Yes | Yes | Yes | Yes | Yes | Yes | Yes | Yes | Yes | Yes | Yes | Yes | Yes | Yes | Yes | No | NA | NA | NA |  | NA | NA | Yes | Yes | Yes |  | Yes | Yes |  | Yes | Yes | Yes |
| 41 | Jafari et. al, 2024 | Yes |  | Yes |  | Yes | Yes |  | Retro | Yes | Yes | Yes | Yes | Yes | No | Yes | Yes | Yes | Yes | Yes | Yes | Yes | Yes | Yes | Yes | Yes | Yes | Yes | Yes | Yes | Yes | Yes | Yes | Yes | No | Yes | Yes | No |  | Yes | No | Yes | Yes | Yes |  | Yes | Yes |  | Yes | Yes | Yes |

| | | | | | | | | | | | | | | | | | | | | | | | | | | | | | | | | | | | | | | | | | | | | | | | | | | | |
|---|---|---|---|---|---|---|---|---|---|---|---|---|---|---|---|---|---|---|---|---|---|---|---|---|---|---|---|---|---|---|---|---|---|---|---|---|---|---|---|---|---|---|---|---|---|---|---|---|---|---|---|
| 1 | Arian et. al, 2022 | Yes |  | Yes |  | Yes | Yes |  | Prosp | Yes | Yes | Yes | Yes | Yes | No | No | Yes | Yes | Yes | Yes | Yes | Yes | NA | NA | Yes | Yes | Yes | Yes | Yes | NA | NA | Yes | Yes | Yes | Yes | Yes | No | No |  | Yes | Yes | Yes | Yes | Yes |  | Yes | Yes |  | Yes | Yes | Yes |
| 2 | Avard et. al, 2022 | Yes |  | Yes |  | Yes | Yes |  | Retro | Yes | Yes | Yes | Yes | Yes | No | No | Yes | Yes | Yes | Yes | Yes | No | NA | NA | Yes | Yes | Yes | Yes | Yes | Yes | Yes | Yes | Yes | Yes | Yes | Yes | No | No |  | Yes | No | Yes | Yes | Yes |  | Yes | Yes |  | Yes | Yes | Yes |
| 3 | Ma et. al, 2021 | Yes |  | Yes |  | Yes | Yes |  | Retro | Yes | Yes | Yes | Yes | Yes | No | No | Yes | Yes | Yes | Yes | Yes | No | NA | NA | Yes | Yes | Yes | Yes | Yes | Yes | Yes | Yes | Yes | Yes | Yes | Yes | No | No |  | Yes | Yes | Yes | Yes | Yes |  | Yes | Yes |  | Yes | No | Yes |
| 4 | Abdulkareem et. al, 2022 | Yes |  | Yes |  | Yes | Yes |  | Retro | Yes | Yes | Yes | Yes | Yes | No | No | Yes | Yes | Yes | Yes | Yes | No | NA | NA | Yes | Yes | Yes | Yes | Yes | Yes | Yes | Yes | Yes | Yes | Yes | Yes | No | No |  | Yes | No | Yes | Yes | Yes |  | Yes | Yes |  | Yes | Yes | Yes |
| 5 | Larroza et. al, 2018 | Yes |  | Yes |  | Yes | Yes |  | Retro | Yes | Yes | Yes | Yes | Yes | No | No | Yes | Yes | Yes | Yes | Yes | No | NA | NA | Yes | Yes | Yes | Yes | Yes | Yes | Yes | Yes | Yes | Yes | Yes | Yes | No | No |  | Yes | Yes | Yes | Yes | Yes |  | Yes | Yes |  | Yes | No | Yes |
| 6 | Liu X et. al, 2023 | Yes |  | Yes |  | Yes | Yes |  | Retro | Yes | Yes | Yes | Yes | Yes | No | No | Yes | Yes | Yes | Yes | Yes | No | NA | NA | Yes | Yes | Yes | Yes | Yes | Yes | Yes | Yes | Yes | Yes | Yes | Yes | No | No |  | Yes | Yes | Yes | Yes | Yes |  | Yes | Yes |  | Yes | Yes | Yes |
| 7 | Frøysa et. al, 2021 | Yes |  | Yes |  | Yes | Yes |  | Retro | Yes | Yes | Yes | Yes | Yes | No | No | Yes | Yes | Yes | Yes | Yes | No | NA | NA | Yes | Yes | Yes | Yes | Yes | Yes | Yes | Yes | Yes | Yes | Yes | Yes | No | No |  | Yes | Yes | Yes | Yes | Yes |  | Yes | Yes |  | No | No | Yes |
| 8 | Durmaz, et al. 2023 | Yes |  | Yes |  | Yes | Yes |  | Retro | Yes | Yes | Yes | Yes | Yes | No | No | Yes | Yes | Yes | Yes | Yes | Yes | NA | NA | Yes | Yes | Yes | Yes | Yes | Yes | Yes | Yes | Yes | Yes | Yes | Yes | No | No |  | Yes | Yes | Yes | Yes | Yes |  | Yes | Yes |  | Yes | Yes | Yes |
| 9 | Raisi-Estabragh, et al, 2023 | Yes |  | Yes |  | Yes | Yes |  | N/A | Yes | Yes | Yes | Yes | Yes | No | No | Yes | Yes | Yes | Yes | Yes | No | NA | NA | Yes | Yes | Yes | Yes | Yes | Yes | Yes | Yes | Yes | Yes | Yes | Yes | No | No |  | Yes | No | Yes | Yes | Yes |  | Yes | Yes |  | Yes | Yes | Yes |
| 10 | Khozeimeh et. al, 2022 | Yes |  | Yes |  | Yes | Yes |  | Prosp | Yes | Yes | Yes | Yes | Yes | No | No | Yes | Yes | Yes | Yes | Yes | No | NA | NA | Yes | Yes | Yes | Yes | Yes | Yes | Yes | Yes | Yes | Yes | Yes | Yes | No | No |  | Yes | No | Yes | Yes | Yes |  | Yes | Yes |  | Yes | Yes | Yes |
| 11 | Di Noto et. al, 2019 | Yes |  | Yes |  | Yes | Yes |  | Retro | Yes | Yes | Yes | Yes | Yes | No | No | Yes | Yes | Yes | Yes | Yes | Yes | NA | NA | Yes | Yes | Yes | Yes | Yes | Yes | Yes | Yes | Yes | Yes | Yes | Yes | No | No |  | Yes | Yes | Yes | Yes | Yes |  | Yes | Yes |  | Yes | No | Yes |
| 12 | Kotu et. al,  2015 | Yes |  | Yes |  | Yes | Yes |  | N/A | Yes | Yes | Yes | Yes | Yes | No | No | Yes | Yes | Yes | Yes | Yes | Yes | NA | NA | Yes | Yes | Yes | Yes | Yes | Yes | Yes | Yes | Yes | Yes | Yes | Yes | No | No |  | Yes | No | Yes | Yes | Yes |  | Yes | Yes |  | No | No | Yes |
| 13 | Rauseo et. at, 2021 | Yes |  | Yes |  | Yes | Yes |  | Prosp | Yes | Yes | Yes | Yes | Yes | No | No | Yes | Yes | Yes | Yes | Yes | Yes | NA | NA | Yes | Yes | Yes | Yes | Yes | Yes | Yes | Yes | Yes | Yes | Yes | Yes | No | No |  | Yes | Yes | Yes | Yes | Yes |  | Yes | Yes |  | Yes | Yes | Yes |
| 14 | Larroza et. al, 2017 | Yes |  | Yes |  | Yes | Yes |  | Retro | Yes | Yes | Yes | Yes | Yes | No | No | Yes | Yes | Yes | Yes | Yes | No | NA | NA | Yes | Yes | Yes | Yes | Yes | Yes | Yes | Yes | Yes | Yes | Yes | Yes | No | No |  | Yes | Yes | Yes | Yes | Yes |  | Yes | Yes |  | No | No | Yes |
| 15 | Baessler et. al, 2017 | Yes |  | Yes |  | Yes | Yes |  | Retro | Yes | Yes | Yes | Yes | Yes | No | No | Yes | Yes | Yes | Yes | Yes | NA | NA | NA | Yes | Yes | Yes | Yes | Yes | Yes | Yes | Yes | Yes | Yes | Yes | Yes | No | No |  | Yes | Yes | Yes | Yes | Yes |  | Yes | Yes |  | Yes | No | Yes |
| 16 | Pujadas et. al, 2023 | Yes |  | Yes |  | Yes | Yes |  | Prosp | Yes | Yes | Yes | Yes | Yes | No | No | Yes | Yes | Yes | Yes | Yes | No | NA | NA | Yes | Yes | Yes | Yes | Yes | Yes | Yes | Yes | Yes | Yes | Yes | Yes | No | No |  | Yes | Yes | Yes | Yes | Yes |  | Yes | Yes |  | Yes | No | Yes |
| 17 | Wang et. al, 2024 | Yes |  | Yes |  | Yes | Yes |  | Retro | Yes | Yes | Yes | Yes | Yes | No | No | Yes | Yes | Yes | Yes | Yes | Yes | Yes | Yes | Yes | Yes | Yes | Yes | Yes | Yes | Yes | Yes | Yes | Yes | No | Yes | No | No |  | Yes | No | Yes | Yes | Yes |  | Yes | Yes |  | Yes | Yes | Yes |
| 18 | Vande Berg et. al, 2024 | Yes |  | Yes |  | Yes | Yes |  | N/A | Yes | Yes | Yes | Yes | Yes | No | No | Yes | Yes | Yes | Yes | Yes | Yes | Yes | Yes | Yes | Yes | Yes | Yes | Yes | Yes | Yes | Yes | Yes | Yes | No | Yes | No | No |  | Yes | No | Yes | Yes | Yes |  | Yes | Yes |  | Yes | Yes | Yes |
| 19 | Deng et. al, 2024 | Yes |  | Yes |  | Yes | Yes |  | Retro | Yes | Yes | Yes | Yes | Yes | No | Yes | Yes | Yes | Yes | Yes | Yes | Yes | Yes | Yes | Yes | Yes | Yes | Yes | Yes | Yes | Yes | Yes | Yes | Yes | Yes | Yes | No | No |  | Yes | Yes | Yes | Yes | Yes |  | Yes | Yes |  | Yes | Yes | Yes |

* Indicate page and/or line number for each checklist item that is present. NA = not applicable.
